# Comparison of Filtering Methods for the Modeling and Retrospective Forecasting of Influenza Epidemics

**DOI:** 10.1371/journal.pcbi.1003583

**Published:** 2014-04-24

**Authors:** Wan Yang, Alicia Karspeck, Jeffrey Shaman

**Affiliations:** 1 Department of Environmental Health Sciences, Mailman School of Public Health, Columbia University, New York, New York, United States of America; 2 Climate and Global Dynamics Division, National Center for Atmospheric Research, Boulder, Colorado, United States of America; Imperial College London, United Kingdom

## Abstract

A variety of filtering methods enable the recursive estimation of system state variables and inference of model parameters. These methods have found application in a range of disciplines and settings, including engineering design and forecasting, and, over the last two decades, have been applied to infectious disease epidemiology. For any system of interest, the ideal filter depends on the nonlinearity and complexity of the model to which it is applied, the quality and abundance of observations being entrained, and the ultimate application (e.g. forecast, parameter estimation, etc.). Here, we compare the performance of six state-of-the-art filter methods when used to model and forecast influenza activity. Three particle filters—a basic particle filter (PF) with resampling and regularization, maximum likelihood estimation via iterated filtering (MIF), and particle Markov chain Monte Carlo (pMCMC)—and three ensemble filters—the ensemble Kalman filter (EnKF), the ensemble adjustment Kalman filter (EAKF), and the rank histogram filter (RHF)—were used in conjunction with a humidity-forced susceptible-infectious-recovered-susceptible (SIRS) model and weekly estimates of influenza incidence. The modeling frameworks, first validated with synthetic influenza epidemic data, were then applied to fit and retrospectively forecast the historical incidence time series of seven influenza epidemics during 2003–2012, for 115 cities in the United States. Results suggest that when using the SIRS model the ensemble filters and the basic PF are more capable of faithfully recreating historical influenza incidence time series, while the MIF and pMCMC do not perform as well for multimodal outbreaks. For forecast of the week with the highest influenza activity, the accuracies of the six model-filter frameworks are comparable; the three particle filters perform slightly better predicting peaks 1–5 weeks in the future; the ensemble filters are more accurate predicting peaks in the past.

## Author Summary

Influenza, or the flu, is a significant public health burden in the U.S. that annually causes between 3,000 and 49,000 deaths. Predictions of influenza, if reliable, would provide public health officials valuable advanced warning that could aid efforts to reduce the burden of this disease. For instance, medical resources, including vaccines and antiviral drugs, can be distributed to areas in need well in advance of peak influenza incidence. Recent applications of statistical filtering methods to epidemiological models have shown that accurate and reliable influenza forecast is possible; however, many filtering methods exist, and the performance of any filter may be application dependent. Here we use a single epidemiological modeling framework to test the performance of six state-of-the-art filters for modeling and forecasting influenza. Three of the filters are particle filters, commonly used in scientific, engineering, and economic disciplines; the other three filters are ensemble filters, frequently used in geophysical disciplines, such as numerical weather prediction. We use each of the six filters to retrospectively model and forecast seasonal influenza activity during 2003–2012 for 115 cities in the U.S. We report the performance of the six filters and discuss potential strategies for improving real-time influenza prediction.

## Introduction

Influenza exacts an enormous toll on human health and economic well-being. Annually, it leads to an average of 610,000 life-years lost, 3.1 million hospitalization days, 31.4 million outpatient visits, and a total economic cost of $87.1 billion in the United States [1]. This burden might be reduced should routine and reliable predictions of influenza outbreaks become available, provided prediction lead times are sufficient to allow the distribution of mitigation and intervention resources. Recent work has shown that influenza outbreaks can be accurately predicted with mathematical models of influenza transmission dynamics that have been recursively optimized using real-time observations of influenza incidence and data assimilation methods [2]–[5]. These findings indicate that infectious disease forecasting is achievable; however, much work remains to be done testing, validating and improving these prediction systems.

In this work, we build on our initial influenza prediction efforts [4], [6], [7] and use a Susceptible-Infectious-Recovered-Susceptible (SIRS) model to explore how the choice of optimization method affects estimates of influenza activity and forecast performance. The SIRS model is comprised of state variables (e.g., number of susceptible persons, *S*), which document the evolution of conditions within the simulated population, and parameters (e.g., infectious period, *D*), which represent biological properties inherent to a given influenza strain and host population and which also can vary from region to region and season to season. The ability of a dynamic influenza model to make accurate predictions depends not only on the fidelity with which the model represents real-world transmission dynamics, but also on the appropriate specification of model parameters and the accuracy of model state variable estimation at the start of a forecast, i.e. the initial conditions. Consequently, it is important for prediction that model parameters and initial conditions be well specified.

Data assimilation, or filtering, methods can be used, in conjunction with the SIRS model and observations of influenza incidence, to estimate the state variable conditions and infer the model parameters. Data assimilation methods use the observations to recursively inform and train the model so that current conditions are better depicted and evolving outbreak characteristics (i.e. the trajectory of the epidemic curve) are better matched. The SIRS model with inferred parameters and updated state variables, can then be propagated into the future to make a more accurate and reliable forecast.

A variety of data assimilation methods exist. The effectiveness of a particular method depends on the structure and dimension of the model to which it is applied, the quality of observations, and the form and limitations of that particular data assimilation method. Consequently, for a given model and application, it can be beneficial to compare a number of data assimilation methods in order to determine whether a more effective method exists. Here, we perform such a comparison by implementing and evaluating six different data assimilation methods using the same SIRS model and observations of influenza incidence. The accuracy and reliability of state variable and parameter estimation, as well as outbreak prediction, using each of these methods is then assessed.

In the Materials and Methods section we present an overview of the data, the SIRS model, the general principle of data assimilation, and each of the specific data assimilation methods tested in this study. We first validate each method using a model-synthesized influenza outbreak. We then use each model-filter framework in conjunction with historical incidence time series from seven influenza epidemics during 2003–2012 for 115 cities in the United States. State variable and parameter estimates are compared and retrospective forecast accuracy is assessed.

## Materials and Methods

### Data

#### (1) Observational estimates of influenza incidence

Regional influenza activity is monitored as influenza like illness (ILI) by the U.S. Centers for Disease Control and Prevention (CDC) [8]. The CDC defines ILI is as a fever (temperature of 100°F [37.8°C] or higher) and cough and/or sore throat without a known cause other than influenza. The ILI definition is not specific for influenza, because many other respiratory diseases manifest with similar symptoms. A more precise diagnosis comes from the World Health Organization and National Respiratory and Enteric Virus Surveillance System, which tests for the presence of influenza virus in samples collected from patients presenting with ILI. Combining the ILI surveillance data with this viral isolation information gives a more specific representation of influenza activity [9].

Google Flu Trends (GFT) data [10] provide weekly, real-time ILI estimates within the U.S. at national, regional, and municipal resolutions. The GFT ILI estimates are derived from internet search query activity and a statistical model that has been retrospectively calibrated to CDC weekly ILI data (see [10] for details). In the continental U.S., these data are provided at the municipal scale for up to 115 cities [7]. In our previous studies [6], [7], we multiplied each weekly municipal GFT ILI estimate by its CDC census division regional influenza viral isolation rate, to generate a near real-time estimate of municipal influenza infection per 100,000 patient visits, termed ILI+. Here we use this same weekly ILI+ metric for modeling, retrospective forecast, and the comparison of data assimilation methods.

It should be noted that ILI data are collected as numbers of infected persons per patient-doctor visit. These data not only miss the infections of persons not seeking clinical assistance but also do not provide a true estimate of incidence rate, which is more directly related to the force of infection in both the real world and model simulations. Further, the ILI observations are themselves error-laden. While these errors pose an important challenge to accurate forecast of influenza, data assimilation methods are equipped to deal with imperfect observations by making explicit estimates of the size of the error associated with the uncertainties in influenza data.

#### (2) Observation error variance

For this study, we assumed the observed variable, i.e. the model counterpart of ILI+, has a Gaussian distribution centered at the observation (i.e., the ILI+ observation was assumed to be the mean) [7]. The variance of each observation, i.e., ILI+ observation error variance (OEV), was calculated per [4], [7]. Specifically, the OEV for week *k*, (*OEV_k_*) was defined as
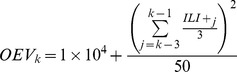
(1)where *ILI+_j_* is the ILI+ estimate for week *j*. Equation 1 indicates that *OEV_k_* is proportional to the average ILI+ estimate during the preceding 3 weeks. This same Gaussian distribution was adopted for all 6 filters tested in this study.

### SIRS model

The model used for this study is a perfectly-mixed, absolute humidity-driven SIRS construct [4]. We use this model as its ability to generate accurate and reliable real-time influenza forecasts has been demonstrated [7]. The SIRS model equations are:
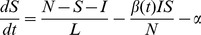
(2)

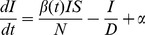
(3)where *S* is the number of susceptible people in the population, *t* is time in years, N is the population size, *I* is the number of infectious people, N – *S* – *I* is the number of resistant individuals, *β(t)* is the transmission rate at time *t*, *L* is the average duration of immunity, *D* is the mean infectious period, and *α* is the rate of travel-related import of influenza virus into the model domain. The basic reproductive number, which is the average number of secondary infections arising from a primary case in a fully susceptible population at time *t* is related to the transmission rate through the expression *R_0_(t)* = *β(t)D*.

Absolute humidity (AH) modulates transmission rates within this model by altering *R_0_(t)* through an exponential relationship similar to how AH has been shown to affect both influenza virus survival and transmission in laboratory experiments [11]:
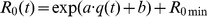
(4)where *a* = −180, *b* = log(*R_0max_−R_0min_*), *R_0max_* and *R_0min_* are the maximum and minimum daily basic reproductive number, respectively, and *q(t)* is the specific humidity, a measure of AH, at time *t* (see Text S1 for details). The value of *a* is estimated from the laboratory regression of influenza virus survival upon AH [12].

The model was run using a stochastic Markov chain formulation in which individuals are treated as discrete entities, i.e. all state variables are whole numbers. Transitions between model states (i.e. susceptible, infectious, recovered) were simulated as a Poisson process with expected rates determined from Equations 2 and 3.

### General setup of the SIRS model-filter frameworks

Bayesian inference methods compute the posterior (i.e., the conditional distribution given the observations) of a parameter or state variable based on the prior (i.e., the probability distribution regardless of the observations) and the likelihood (i.e., the conditional distribution of the observations given the parameters or state variables). Here, we define the model ‘state’ as all variables within the model-filter framework, including both the variables (*S* and *I*) and the model parameters (*L*, *D*, *R_0max_*, and *R_0min_*.). A Monte Carlo approach, in which multiple representations of the system are treated as samples from the prior and posterior distributions, can be adopted for performing data assimilation. Each system representation, termed a particle or ensemble member, consists of a different combination of the model state. The data assimilation process begins with a suite of unique particles or ensemble members (e.g. 10,000 or 300 model simulations). The filtering procedure includes repeated short-term (e.g., 1 week) prediction-update cycles; the prediction propagates the particles/ensemble forward according to the SIRS model equations, and the update, triggered by arrival of a new observation record, assimilates that observation to refine the estimate of the state. These updates enable optimization of the model state to characterize the ongoing observed epidemic.

Specifically, the SIRS model is propagated through time *k* = 1, 2, …, per Eqs. 2 and 3, and makes a prediction of the new state, ***x_k_***. This state is related to the latest observation of ILI+, ***z_k_***, through a normally distributed likelihood function:
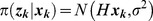
(5)where *H* is the mapping from state space to observation space, and *σ^2^* is the observation error variance, as defined in Eq. 1. The filter is then used to find the posterior, i.e. *π*(***x_k_***|***z_1:k_***), given a sequence of observations, the likelihood, and the model prediction. This problem is solved by Bayes' rule:
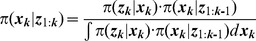
(6)This posterior estimate then replaces the prior state prediction, the model is propagated forward to the next observation and the process is repeated.

All filter methods use these prediction-update cycles; however, different filter methods make different assumptions. For instance, in the application presented here, the parameters of the SIRS model (*L*, *D*, *R_0max_*, and *R_0min_*) are formulated as constants (i.e. Eqs. 2–4), and these constant parameters are inferred through the prediction-update cycles of the filter process. However, while the same SIRS model construct is used, different filter methods handle parameter estimation differently. Some of the filters revise their parameter estimates at each data assimilation checkpoint, and the entire filtering process is done with a single round of integration through the observed time series. Other filters use multiple rounds of integration through the observed time series and optimize the model parameters based on the entire observation record (i.e., from the beginning to the end of simulation).

Some of the filters are also built upon specific assumptions that enable the simple solution of the filtering algorithm. For instance, two of the ensemble filters we test here assume that the range of state space conditions depicted by the ensemble conforms to a Gaussian distribution, which makes these filters sub-optimal for non-linear systems. Other filters, such as particle filters make no assumption about the form of the distributions, which, in theory, makes them more suitable for nonlinear systems. Nevertheless, both ensemble and particle filters have been successfully used to describe non-linear systems [2], [13]–[17]. For a weakly nonlinear system, such as the SIRS model used here, it is difficult to know in advance whether the linear, Gaussian assumption that confers the ensemble filters their computational tractability leads to degraded performance relative to the particle filters.

The particle filters [16], [18]–[20] and ensemble filters [13], [14], [21], which we will be exploring here, are data assimilation methods that have been applied in infectious disease modeling, inference, and forecasting [2], [4], [17], [22]. The maximum likelihood via iterated filtering (MIF) [16], [22] method, based on particle filtering, has been applied to study the transmission of cholera [16], [23], measles [24], and influenza [25]. Rasmussen and colleagues [17] applied the particle Markov chain Monte Carlo (pMCMC) [20], an algorithm combining particle filtering and MCMC methods, to simulate influenza prevalence data (generated from an SIR model) and showed that the pMCMC was able to recreate synthetic time series with parameter estimates close to those used to generate the synthetic data. The ensemble adjustment Kalman filter (EAKF) has been used to forecast observed seasonal influenza outbreaks and was able to produce accurate retrospective and real-time predictions of epidemic peak timing [4], [7].

Each of the six filters used in this study is described in turn below. We focus on presenting the principal features of each filter. For technical details, we refer the readers to the original publications. Implementation details are available in Text S1; the code for the six influenza forecast frameworks written in R (http://www.R-project.org) is available from our website: http://cpid.iri.columbia.edu/Refs.html.

### Particle Filters

#### (1) Basic particle filter (PF)

The basic particle filter (PF) used in this study is primarily based on Algorithm 3 in [18]. The PF populates state space with a set of discrete particles and makes no assumption of the overall distribution of these particles. Per the core prediction-update cycle, particles are propagated forward in time using the SIRS model, and each week, ILI+ observations are used to weight each particle according to its likelihood.

#### (2) MIF

In contrast to the basic PF, which performs state and parameter estimation at each data-assimilation checkpoint during a single round of integration through the observed time series, the MIF, proposed by Ionides et al. [16], [22], makes state and parameter estimates based on multiple rounds of particle filtering, i.e. multiple iterations, through the time series of observations. The assimilated observed time series record consists of all observations available; that is, for fitting the epidemic curve, it includes weekly ILI+ records of an entire season, and for the forecasts, it includes ILI+ records up to the latest observation before the start of forecast. Within each round of filtering, i.e., each iteration, a PF is used, which allows the filter to explore parameter space. The posterior estimate of parameters achieved when all available observations have been assimilated is then used to inform the prior for the next iteration. Specifically, the new prior of parameters takes on a multinormal distribution with the means of the posterior from the last iteration and a prescribed narrowed covariance matrix (see [16], [22] and Text S1). This procedure updates the parameter estimates in the direction of increasing likelihood such that it finally converges to the maximum.

#### (3) pMCMC

The pMCMC used in this study is based on the particle marginal Metropolis-Hastings sampler in [20]. This algorithm decouples the state variables and model parameters, applying an MCMC method to constrain the model parameters while the PF is used between MCMC iterations to estimate the state given a set of parameters proposed by the MCMC step. Acceptance of a parameter proposal is based on the joint likelihood over the entire assimilated observation time series (as for the MIF), per the Metropolis-Hastings algorithm. The set of parameters proposed for each MCMC proposal is used throughout the simulation and for all particles; therefore the parameters are stationary.

### Ensemble filters

The ensemble filter variants described below differ only in how the observed variable is updated. Two of the ensemble filters we describe below, the ensemble Kalman filter (EnKF) and the EAKF, assume that the prior and posterior are Gaussian and that the function mapping from the state to the observational space is a linear operator [13], [14], [26].

#### (1) EnKF

In the observation space, the EnKF [14] computes the posterior ensemble as a weighted average of the prior and the measurement according to the following equation:
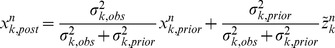
(7)where, for week *k*, 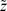 is the ILI+ measurement plus Gaussian random noise with variance *OEV_k_*, *x* is the observed variable (i.e., the model counterpart of *z*), *σ^2^* is the variance, the superscript *n* denotes the *n*th ensemble member, and the subscripts *obs*, *prior*, and *post*, denote the observation, prior, and posterior, respectively. Note 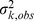 is the same as *OEV_k_* in Eq. 1.

The unobserved variables and parameters are adjusted based on the increment of the observed variable. Specifically, the increment of the *n*th ensemble member for the *m*th unobserved variable or parameter of the state, 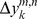, i.e., the posterior minus the prior, is calculated as:
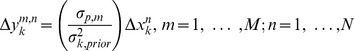
(8)where *σ_p,m_* is the prior covariance of the observed and the *m*th unobserved variable or parameter of the state, 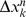 is the increment of the observed variable (i.e. 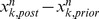), *M* is the number of variables of the state, and *N* is the ensemble size.

#### (2) EAKF

Unlike the EnKF, the EAKF algorithm, based on [4], [21], [27], does not add Gaussian noise to the ILI+ measurement. To form the posterior ensemble, the EAKF deterministically adjusts each ensemble member towards the ensemble mean (Eq. S1) such that the posterior variance is identical to what is predicted by Bayes' theorem (assuming Gaussian distributions). The unobserved variables and parameters are then adjusted per Eq. 8.

#### (3) RHF

The third ensemble filter, the rank histogram filter (RHF; [13]), does not restrict the form of the prior, observation, or posterior, a feature that makes it attractive for use in nonlinear systems, which do not, in general, follow normal distributions. However, like the other two ensemble filters, it shares the assumption that there is a linear relationship between an observation and all the model state variables. In this aspect, it does not completely resolve the problem of being suboptimal for non-linear systems. In the RHF [13], the ensemble members are used to empirically construct a continuous density distribution of the prior, which is then multiplied by the likelihood function to determine the posterior.

### Model validation

A mock time series of influenza prevalence (*I*) and number of susceptible persons (*S*) was generated using the SIRS model for a population of 100,000 with an initial susceptible rate of 60%. The parameters used were the same as in [4], i.e., *L* = 3.86 y, *D* = 2.27 d, *R_0max_* = 3.79, and *R_0min_* = 0.97; historical AH conditions from September 29, 1972 to May 14, 1973 for New York City were used as the humidity forcing data. We refer to this mock time series as the synthetic ‘truth’.

Random Gaussian noise (zero mean and a variance of 10000) was then added to the synthetic truth to mimic error-laden observations and used as the initial test data for all six filters. Specifically, the SIRS model was run in conjunction with each of the six filters using the synthetic time series as observations, and final state variable and parameter estimates were compared with the truth. For each filter, a suite of particles or ensemble members was employed (see Text S1 for details). Initial variable and parameter values for each particle or ensemble member were chosen randomly from a distribution of possible values, as in [4]. To assess the effects of initial particle or ensemble member random selection on the final state estimates, each filter was run 25 times.

### Modeling historical ILI+ time series

The six filtering methods were then applied to the SIRS model and weekly ILI+ for the 2003–04 through 2011–12 seasons for 115 cities in the U.S. [7]. We excluded the 2008–09 and 2009–10 seasons, which spanned the 2009 pandemic, to focus on seasonal influenza.

All model-filter frameworks were run for a single season at a time, as opposed to continuously from 2003 to 2012. Briefly, at the beginning of each season (i.e., Week 40 of each year, before influenza activity), the system was initialized with randomly selected values of all model state variables and parameters; the system was then propagated forward per the SIRS model and weekly ILI+ observations were assimilated up to the end of each season (i.e., Week 39 of the next year). Due to the intense computational demand, especially for the MIF and pMCMC, this process was repeated 5 times for each combination of filter, city and season. For instance, five separate MIF runs were performed for Chicago during the 2004–05 season. We calculated the correlation and root mean squared (RMS) error between the model simulated time series and the corresponding historical ILI+ time series, for each run.

### Retrospective forecasting

Retrospective forecasting was performed for the 115 cities during the seven aforementioned influenza epidemics. Each forecast framework was run for a training period of 3–28 weeks beyond Week 40 of each year. The training period assimilates observations (i.e., 3–28 ILI+ weekly records) to estimate the state (including *I* and *S*, and all model parameters) up to the point of forecast. The forecast is then generated by integrating the SIRS model forward with the last state estimate from the training period up to Week 39 of the next year. Thus, for each combination of filter, city and influenza season, 26 different weekly forecasts were generated—a forecast following 3 weeks training, a forecast following 4 weeks training, etc. This process was repeated 5 times to assess the effects of initial particle or ensemble member random selection.

Forecast accuracy was evaluated for prediction of the peak week of each influenza outbreak, i.e., the week with the highest observed ILI+. For each run, each particle (for the particle filters) or ensemble member (for the ensemble filters) generates a prediction; the collective prediction of the whole run is taken as the value (i.e., the week of peak) most frequently predicted. We refer to this metric as mode predicted peak. Mode predicted peaks occurring within ±1 wk of the observed ILI+ peak were deemed accurate.

## Results

### Synthetic runs

**Figure 1:**
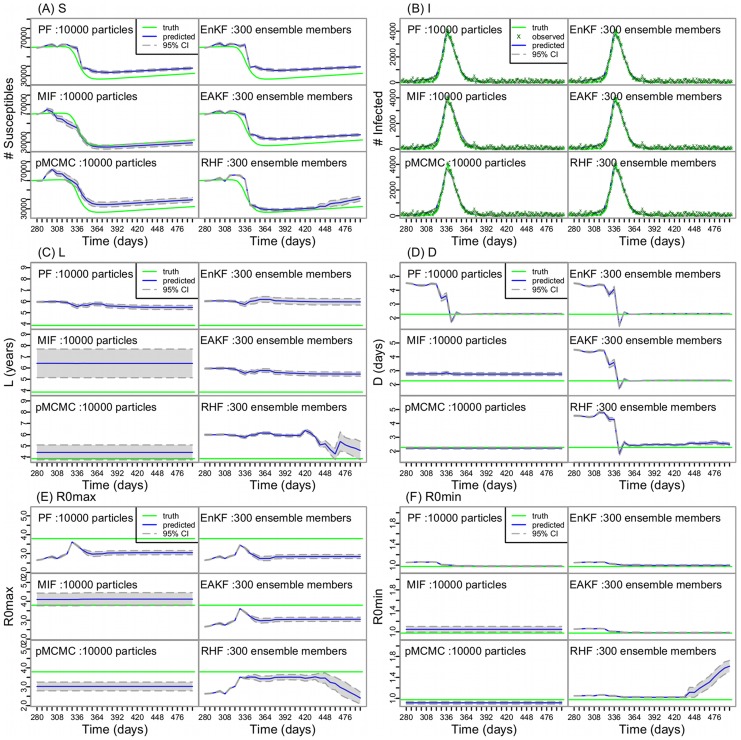
State estimation using the six filters and synthetic ‘truth’. The model-filter framework includes two model state variables and four model parameters: (A) the number of susceptible persons *S*, (B) the number of infected persons *I*, (C) the immunity period *L*, (D) the infectious period *D*, (E) the maximum reproductive number *R_0max_*, and (F) the minimum reproductive number *R_0min_*. The synthetic ‘truth’ for *I* and *S* was generated with the SIRS model, for a population of 100,000, with fixed parameters: *L* = 3.86 y, *D* = 2.27 d, *R_0max_* = 3.79, and *R_0min_* = 0.97; each model-filter framework was run repeatedly 25 times, with the same set of test data (i.e., synthetic ‘truth’ for the *I* time series plus random noise, shown as ‘x’ points in (B)). The green lines are the synthetic ‘truth’, blue lines are the mean trajectory over the 25 runs, and the grey areas around them delineate the 95% confidence interval.

Figure 1 shows the time series of state variables (i.e., susceptible and infectious levels, *S* and *I*) and four model parameters inferred from each filter. All filters produced time series of *I* and *S* that are highly correlated to the ‘truth’ (*r*>0.99 for *I*, and *r*>0.94 for *S*, averaged over 25 runs of each filter).

For the estimates of model parameters (e.g., infectious period *D*), the basic PF and ensemble filters adjust their parameter estimates over a single integration through the observed time series, whereas the MIF and pMCMC estimates are shown, respectively, for their final and best integrations through the observed time series (Figure 1). As the epidemic unfolds, the parameters inferred from the basic PF, EnKF, EAKF, and RHF gradually approach the ‘truth’. These filters were able to locate the ‘truth’ by the peak of the epidemic. In contrast, parameters inferred from the MIF and pMCMC were the estimates taken from the final iteration or best simulation. By the outbreak peak, estimates for key epidemiological parameters, such as the infectious period, *D*, and the maximum daily basic reproductive number, *R_0max_*, made by the six filters were comparable and all close to the ‘truth’ (Figure 1). These results indicate that all six model-filter frameworks are able to recreate the synthetic ‘truth’ by the peak of the outbreak as well as generate reasonable parameter estimates despite the random error added to the synthetic *I* time series.

### Simulation of historical ILI+ time series for 115 cities in the U.S

**Figure 2:**
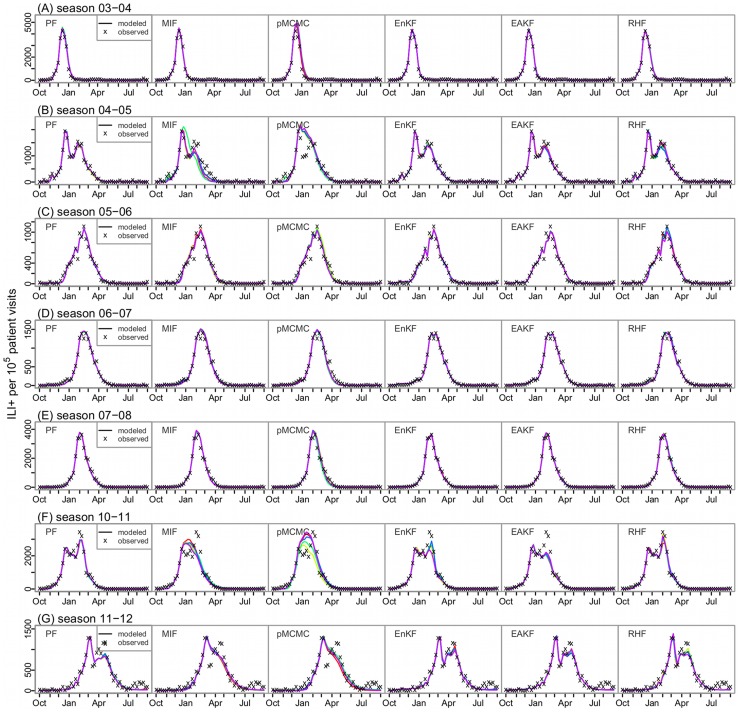
ILI+ time series generated from the six filters for New York City. Simulations are shown for seasons (A) 2003–04, (B) 2004–05, (C) 2005–06, (D) 2006–07, (E) 2007–08, (F) 2010–2011, and (G) 2011–12 (excluding the pandemic seasons). Each model-filter framework was run repeatedly 5 times; each colored line represents one run; the ‘x’ points are the observed weekly ILI+ data.

We then applied these model-filter frameworks to municipal weekly ILI+ time series collected during the 2003–04 through 2011–12 influenza epidemics, excluding the 2008–09 and 2009–10 pandemic seasons, for 115 cities in the U.S. Overall, all the model-filter frameworks were able to generate epidemic curves that corresponded well with the historical ILI+ time series. The correlation coefficients for the six model-filter frameworks over all seasons and cities are (in descending order): 0.991 (95% CI: 0.9907–0.9913, EnKF), 0.990 (0.9896–0.9905, RHF), 0.989 (0.989–0.990, EAKF), 0.985(0.984–0.986, PF), 0.966 (0.965–0.967, pMCMC), and 0.966 (0.964–0.967, MIF). Figure 2 shows the epidemic curves estimated by the six filters for New York City over the seven flu seasons. For seasons with a unimodal outbreak (i.e., 2003–04, 2005–06, 2006–07, 2007–08), all filters were able to fit the outbreaks well. For seasons with a multimodal outbreak (i.e., 2004–05, 2010–11, and 2011–12), performance differed among the filters. The basic PF and the three ensemble filters, with parameters adjusted at each data-assimilation checkpoint during a single integration, were more capable of matching multimodal outbreaks.

**Figure 3:**
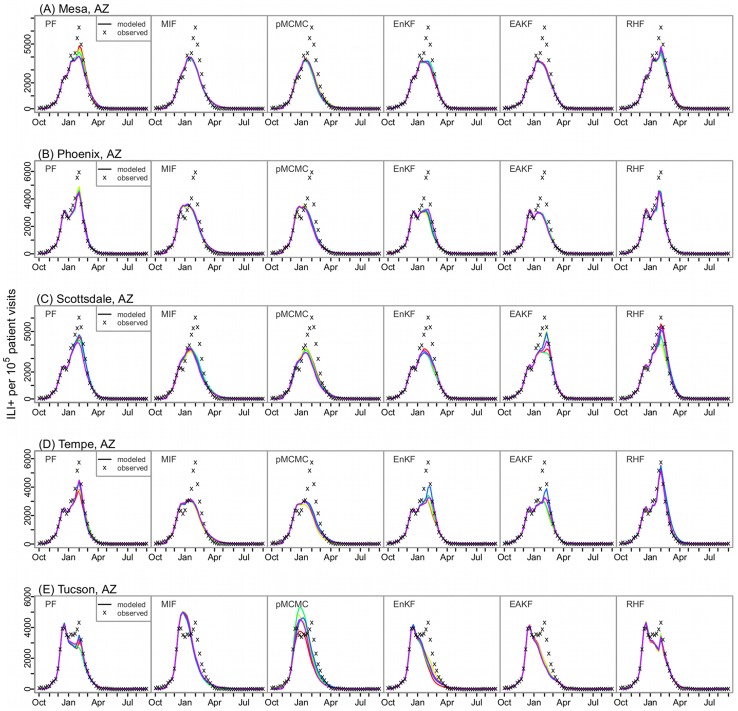
Fitting multimodal outbreaks. The model-filter frameworks were tested with historical ILI+ time series collected in the 2010–11 season from 5 cities in Arizona: (A) Mesa, (B) Phoenix, (C) Scottsdale, (D) Tempe, and (E) Tucson; all ILI+ times series had multiple peaks of varying magnitudes; each model-filter framework was run repeatedly 5 times; each colored line represents one run; the ‘x’ points are the observed weekly ILI+ data.

Performance of the six filters differed most in representing multimodal outbreaks with peaks of similar magnitudes. For instance, cities in Arizona had an A(H3) outbreak followed by an A(2009H1N1) outbreak of comparable (Figure 3E) or greater (Figure 3A–D) magnitude during the 2010–11 season. As shown in Figure 3, the MIF and pMCMC generated a single peak lying between the two consecutive outbreaks; the EnKF and EAKF were able to generate two peaks to some extent matching the observations (Figure 3A–D); the RHF and the basic PF were able to generate time series most closely matching the observations. The RHF and the basic PF construct posterior distributions based on the likelihood without strict constraints on the prior (i.e., unlike the Gaussian distribution for the EnKF and EAKF) or the parameters (e.g., parameters are not adjusted by the pMCMC within a single integration through the observed record). This feature allows the RHF and basic PF to capture erratic changes in the ILI+ time series due to factors such as a switch of circulating strain. Figure S2 shows that the ensemble filters and the basic PF, in particular the RHF and the basic PF, were able to adjust key epidemiological parameters during a single integration to reflect sudden changes in the observed epidemic curve. In contrast, the MIF, which has parameter estimates that shift very little during its final iteration, and the pMCMC, which uses the stationary parameter estimates from its best proposal, both tended to generate outbreaks with a single peak and did not represent multimodal outbreaks as well.

**Figure 4:**
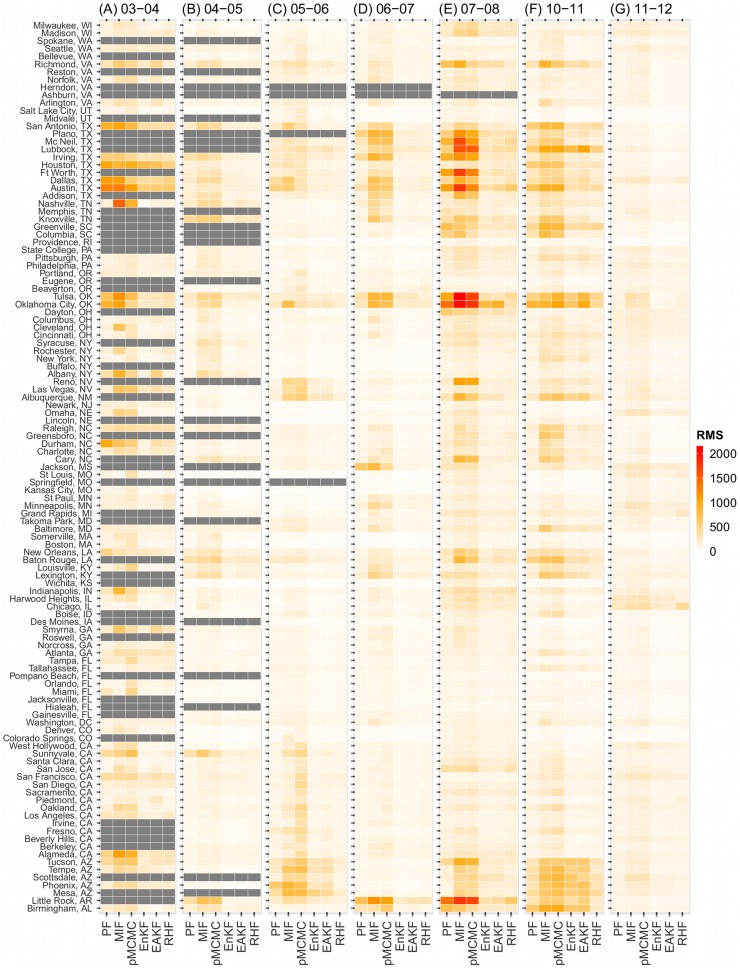
The Root Mean Squared (RMS) error of the six model-filter frameworks used to simulate historical ILI+ for 115 major U.S. cities during the 2003–2012 flu seasons. Each model-filter framework was run repeatedly 5 times; the RMS error between the predicted and observed ILI+ time series was calculated for each run; the color of each rectangle, corresponding to each city (y-axis) by each filtering framework (x-axis), indicates the average RMS error over the 5 repeated runs for epidemic seasons (A) 2003–04, (B) 2004–05, (C) 2005–06, (D) 2006–07, (E) 2007–08, (F) 2010–2011, and (G) 2011–12.

To evaluate the filters more objectively, we calculated the RMS error of each simulation. Table 1 shows the average RMS errors over each flu season and all seasons for each filter and Figure 4 color-maps the average RMS over repeated runs for each filter for the 115 cities over seven flu seasons. Three patterns are evident from Figure 4: (1) The ensemble filters and basic PF performed better and hence had lower RMS errors (lighter colors) in general. Of the top 10% of all filtering runs (21,750 in total) with the lowest RMS errors (referred to as the best runs hereafter), 28.7%, 25.0%, 19.0%, and 12.4% were produced with the EnKF, EAKF, RHF, and PF, respectively. Of the top 10% with the highest RMS errors (referred to as the worst runs hereafter), 34.1% and 34.0% were produced with the MIF and pMCMC, respectively, while only 4.9%, 5.4%, and 8.4% were produced by the RHF, EnKF, and EAKF, respectively. (2) Some seasons, for instance, 2003–04, 2007–08, and 2010–11, were more difficult to simulate and had higher RMS errors (darker colors in the corresponding columns). This tendency is seen for all filters (Figure 4A, E, and F, and Table 1) although more so for the three particle filters. Seasons 2007–08 and 2010–11 make up 50% of the worst runs. (3) Across all years, cities in 3 states, Texas (10 cities), Arizona (5 cities), and Oklahoma (2 cities), had higher RMS errors. These three states made up 28.5%, 13.3%, and 9.0%, respectively, of the worst runs. These cities often experienced multimodal ILI+ outbreaks or higher levels of GFT ILI (e.g. peak ILI+ was 2–4 times levels observed in other seasons/cities). Such characteristics appear to be more problematic for the particle filters, particularly the MIF and pMCMC, whereas the ensemble filters appear more robust in these situations. Similar patterns are also seen when the filters are evaluated for correlation (Figure S3).

**Table 1:** Root Mean Squared (RMS) error of the six filters in each flu season.

Season	PF	MIF	pMCMC	EnKF	EAKF	RHF
2003–04	277.77 (246.75–308.78)	418.09 (372.98–463.19)	373.08 (349.63–396.54)	203.87 (187.61–220.12)	163.31 (149.42–177.21)	193.61 (179.34–207.88)
2004–05	112.83 (105.33–120.32)	189.09 (176.28–201.9)	184.25 (172.88–195.63)	101.71 (96.73–106.7)	88.71 (84.5–92.91)	97.56 (93.03–102.09)
2005–06	117.21 (109.4–125.03)	182.99 (168.86–197.12)	205.52 (192.29–218.75)	104.26 (98–110.52)	94.71 (88.89–100.54)	86.85 (83.59–90.10)
2006–07	112.56 (103.33–121.8)	211.08 (191.89–230.26)	194.74 (179.44–210.04)	81.79 (77.87–85.71)	74.80 (71.34–78.26)	86.97 (83.37–90.57)
2007–08	226.63 (203.98–249.28)	384.82 (348.18–421.46)	339.03 (309.76–368.3)	141.01 (131.73–150.3)	128.37 (120.35–136.4)	147.80 (139.4–156.19)
2010–11	170.40 (157.88–182.91)	300.60 (280.65–320.55)	304.24 (284.34–324.14)	190.74 (175.19–206.29)	170.84 (157.8–183.88)	142.73 (134.27–151.19)
2011–12	106.56 (103.38–109.74)	142.53 (137.37–147.69)	155.15 (149.88–160.43)	87.45 (84.08–90.81)	82.50 (79.3–85.7)	94.69 (88.61–100.77)
All	154.51 (148.79–160.22)	253.51 (244.21–262.8)	244.99 (237.63–252.35)	126.33 (122.54–130.13)	112.53 (109.3–115.76)	117.62 (114.74–120.50)

Each of the six model-filter frameworks was run 5 times to simulate the historical ILI+ time series for 115 U.S. cities during each flu season. RMS error for each run was calculated; the numbers presented are average RMS error and 95% confidence intervals (in parentheses) over all runs and all cities for each model-filter framework.

### Retrospective forecasting

**Figure 5:**
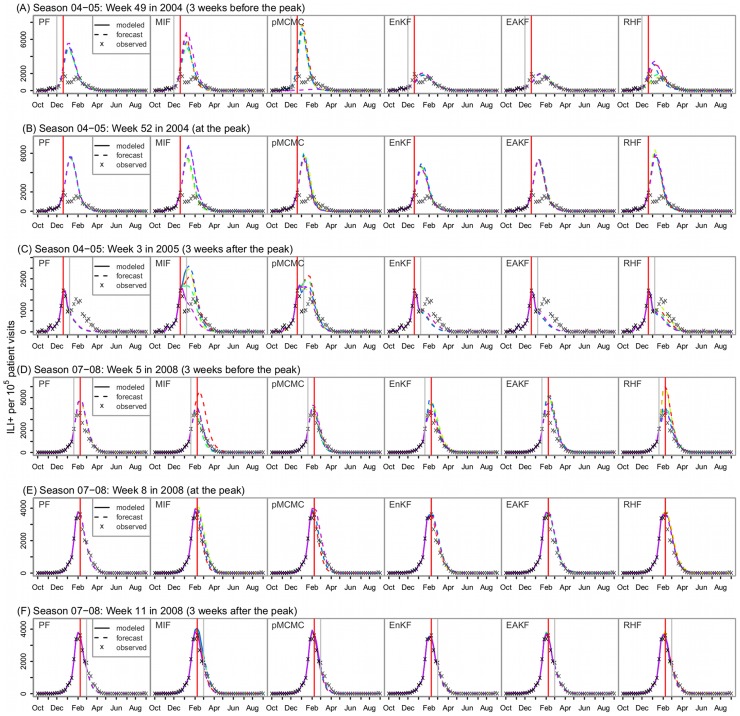
Predicted ILI+ time series for New York City in the 2004–05 (A–C) and 2007–08 seasons (D–F). Solid lines (5 lines from five repeated runs for each filter) are modeled based on observations during the training period, and the dashed lines are the forecasts; Red vertical lines indicate the observed peak, and grey vertical lines mark the week the forecasts are made.

We further tested the performance of the six filters in forecasting influenza outbreaks, retrospectively, for the seven seasons. Each filter was run for a training period, which assimilates all observations up to the week of forecast, makes state estimates reflecting the latest dynamics of the unfolding epidemic, and generates a forecast for the rest of the influenza season by integrating the SIRS model without any further filter constraint. Figure 5 shows the predicted ILI+ time series, both training and forecast, generated at the peak of the influenza outbreak and 3 weeks before or after the peak for New York City in the 2004–05 and 2007–08 seasons. Forecast accuracy with the filters varies. The 2004–05 season had three overlapping outbreaks. For this extremely ‘challenging’ season, even at 3 weeks past the major peak, the MIF and the pMCMC still miss this peak and greatly over-estimate the magnitude of the outbreak; in contrast, the basic PF and the ensemble filters are able to accurately predict the peak and the magnitude of that outbreak (Figure 5 A–C). For the 2007–08 season with a ‘simple’ peak, all filters are able to predict the peak over 3 weeks before the actual event; however, the predicted ILI+ time series made by the three particle filters match the observations marginally better than the ensemble filters (mean RMS error: 330.8 by the particle filters vs. 438.7 by the ensemble filters, 1 sided t-test, *p* = 0.056) (Figure 5 D).

**Figure 6:**
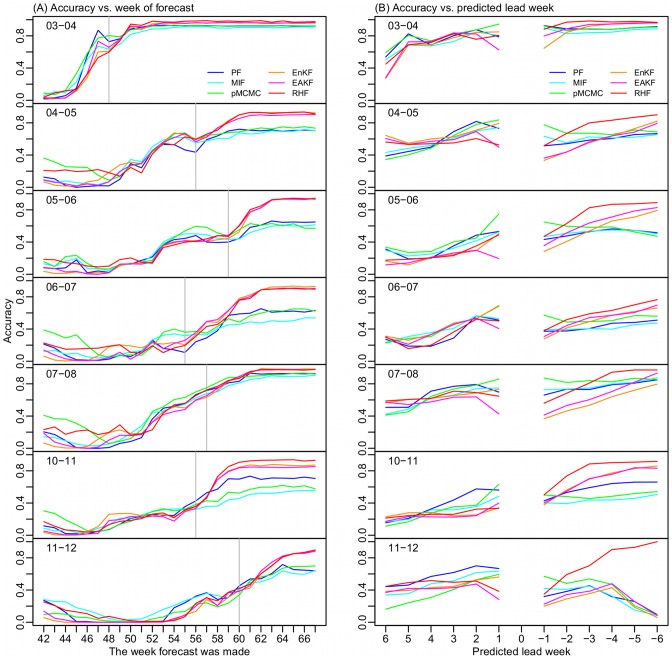
Peak timing prediction accuracy. (A) Accuracy plotted as a function of forecast initiation week; numbers greater than 52/53 weeks are those in the next year, e.g., Week 54 is the first week in 2004 of the 2003–04 season, as 2003 had 53 weeks, and it is the second week in the rest of the seasons; the grey vertical line indicates the peak week most frequently observed among the 115 cities. (B) Accuracy for each predicted lead time with respect to the week of forecast; negative values represent time in the past, e.g., −1 is a peak predicted 1 week in the past. The week 0 predictions are omitted.

Figure 6A shows the accuracy of forecast for peak timing plotted as a function of forecast week (i.e. week of the influenza season). Generally, upon approaching the observed ILI+ peak (gray line), all the filters generate more accurate forecasts. Before the observed peak, forecasts generated using the particle filters appear to have higher accuracy than those generated using the ensemble filters. However, at weeks beyond the actual peak, the ensemble filter forecasts become more accurate. This difference is particularly true for seasons with multimodal outbreaks, e.g, season 2004–05. Accurate prediction of peaks that have already passed is an important characteristic, indicating that the model is not forecasting spurious future influenza incidence.

Figure 6B shows peak timing forecast accuracy for predictions ranging from 6 weeks in the future to 6 weeks in the past (with reference to the time of forecast). We excluded zero-week lead forecasts as most of these predictions are made early in the season (over 50% during the first 8 week forecasts) and are an artifact of filter spin up during pre-outbreak weeks. For forecasts of an outbreak peak 1 to 4 weeks in the future, the three particle filters appear more accurate (57% [41%, 72%] vs. 49% [37%, 63%], 1 sided t-test, *p* = 0.016); for those predicting 1 week in the future or 1 week in the past, the pMCMC is more accurate (70% [67%, 72%] vs. 47% [31%, 64%] for the other five filters, 1 sided t-test, *p* = 0.00097); and for those predicting 2 to 6 weeks in the past, the ensemble filters, particularly the RHF, are more accurate (69% [44%, 90%] vs. 60% [52%, 66%], 1 sided t-test, *p* = 0.011).

A quantification of forecast certainty is critical. When forecast retrospectively, prediction accuracy can be evaluated based on the complete ILI+ record for each flu season, but for real-time forecasts, such an evaluation of accuracy is not possible. It is possible, however, to calibrate prediction accuracy based on the agreement of forecast outcomes. That is, a suite of particles or ensemble members comprises each forecast, and the spread of the collection of these individual forecasts may indicate the confidence of the combined forecast (i.e., the mode in this study). Shaman and Karspeck [4] showed that for the EAKF, decreased ensemble variance coincided with increased forecast accuracy. Similarly, we found that for forecasts with a mode predicted peak 1 to 10+ weeks in the future or 0–5 weeks in the past, the ensemble filters show increasing accuracy as ensemble variance decreased (Figure S4 D–F). In contrast, for the particle filters, this relationship holds for those with the mode 1–3 weeks in the future and 0–5 weeks in the past, but is less obvious for longer lead forecasts (Figure S4 A–C).

**Figure 7:**
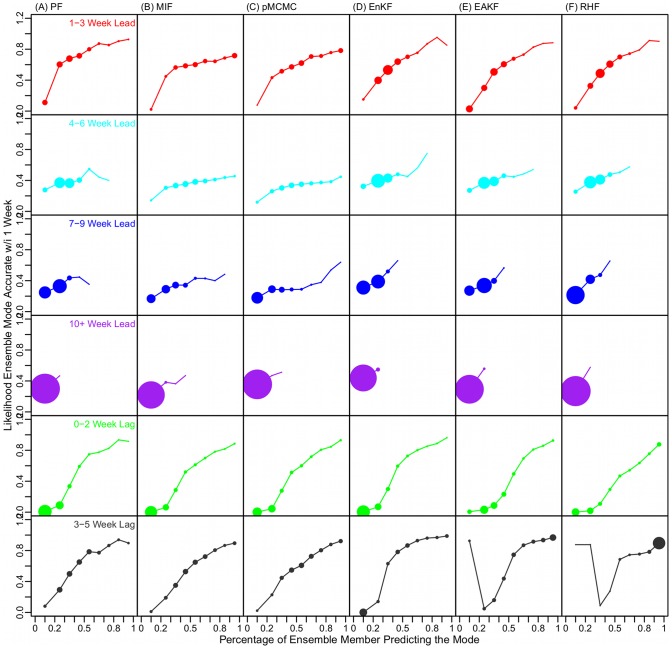
Confidence in the prediction. All forecasts, 565,500 in total, were first categorized according to the mode predicted peak, e.g., 1–3 weeks in the future (the first row) or 3–5 weeks in the past (the last row); within each category, forecasts were further binned by the percentage of ensemble members predicting the mode (PEMPM, e.g., 50–60%) as indicated on the x-axis; the accuracy of forecasts within each bin were then calculated, as shown on the y-axis. The size of the dot in each PEMPM bin indicates the portion of forecasts, within each category, falling into a corresponding bin. Each column (A–F) shows the relationship between the forecast accuracy and the PEMPM for a different filter.

For the particle filters, due to a much larger number of particles (10,000 in this study v. 300 ensemble members for the ensemble filters), more outliers may exist and lead to a larger variance, even for those runs with a narrow kernel spread (e.g., a fat-tailed distribution). An alternate, non-Gaussian, measure of forecast convergence can be made by calculating the percentage of particles/ensemble members predicting the mode (PEMPM). Using PEMPM in place of particle/ensemble member variance, a clearer relationship emerges in which forecast accuracy increases as PEMPM increases (Figure 7). This relationship holds for all the filters, for predictions 1–9 weeks in the future and 0–5 weeks in the past.

## Discussion

The optimal filter method for any dynamic system depends on not only the characteristics of the system, but also the ultimate application. Here we applied six filter methods to the modeling and forecasting of influenza incidence using the same SIRS model. We emphasize that this comparison was performed specifically in the context of modeling and forecasting influenza and is not a general assessment of the tested filters; consequently, the findings may not extend to other systems. Moreover, this comparison did not exhaustively compare all available filter algorithms; more suitable algorithms may yet exist but were not included in this study.

Our findings indicate that the ensemble filters and basic PF did a better job simulating most historical ILI+ time series, while the MIF and pMCMC struggled with multimodal outbreaks. For the forecast of peak timing, the expected accuracies of the six model-filter frameworks were found to be comparable; the particle filters performed slightly better predicting peaks 1–5 weeks in the future; the ensemble filters were better at indicating that the seasonal peak had already occurred. As discussed below, these outcomes are due both to the nature of the ILI+ time series, the SIRS model, and features of each filter.

### Comparison of the filters in simulating historical ILI+ time series

The ensemble filters and basic PF generally simulated historical ILI+ time series better than the MIF and pMCMC. This difference in performance appears to stem from differences in filter design. Specifically, the ensemble filters and the basic PF adjust model parameters continually (at each prediction-update cycle) over the course of a single integration through the observed time series record. In so doing, these filters can flexibly adjust the SIRS model variables and parameters to better depict noisy observations and compensate for the limited behavior of the SIRS model. It is interesting that the ensemble filters, which are often applied to more complex, higher dimension systems for which particle filters collapse [28], are here shown to be adept at handling simulation with a low-dimension, misspecified model.

In contrast, the MIF, over the course of multiple integrations, or iterations, through the observed time series record, converges to a set of parameter estimates that change very little during the final integration (Figure S2). Similarly, the pMCMC uses a single set of time-stationary parameter estimates for each of its multiple integrations through the observed time series, and ultimately selects a best-fitting combination from among these proposals. For both the MIF and pMCMC, the final parameter estimates have been fitted to the entire time series of observations and shift very little (MIF) or not at all (pMCMC) within these final integrations to compensate for model error, such as when the observed ILI+ trajectory deviates strongly from preferred model behavior.

Epidemiological key parameters are often assumed to reflect essential characteristics of the infectious agent and are thus thought to be fixed in time. For instance, the infectious period, *D*, in our simple SIRS model is implicitly the mean of an exponential distribution [29]. In reality, however, influenza outbreak characteristics can change through time, as within population variability, changing observational bias, or the presence of multiple strains can effectively produce changes to key parameters. On the other hand, estimates of influenza incidence, including the ILI+ metric used in this study, are error-laden and do not precisely represent the incidence of disease within a population [6], [30], [31]. Further, this observational error may shift in time. For example, a sudden increase in ILI+ may not be wholly due to a dramatic increase in influenza incidence; rather, it could also reflect increased virulence of the circulating strain leading to more severe symptoms and more outpatient visits, or increased media coverage leading to more web-search activity and higher GFT ILI rates [6]. The parameter estimates based on such data, thus not only reflect the characteristics of the circulating strain (e.g., infectious period *D*), which may be stationary, but also population behavior (e.g., web-search and treatment seeking behavior), which can change over time [6], [7]. The basic PF and the three ensemble filters, by adjusting both the state variables and model parameters at each assimilation checkpoint, and are thus able to match the unfolding epidemic trajectory flexibly. In contrast, the MIF, with parameter estimates that shift very little during its final iteration, and the pMCMC, with its stationary parameter estimates, are not able to incorporate these variations. In the future, the SIRS model structure could be expanded to represent fluctuations in the system parameters. Such model changes may improve the performance of the MIF and pMCMC.

Multimodal outbreaks (i.e., multiple peaks) often result from the co-circulation of multiple influenza strains. Because our SIRS model does not simulate multiple strains, the state variable and model parameter estimates derived from the assimilation process will reflect the aggregate features of those multiple circulating strains. To capture multiple peaks in the ILI+ time series, the filter must promptly catch the changes in the state variables (e.g., *S*, which reflects population immunity level) and model parameters at the switch of the dominant strain. As shown in Figure 3, this adjustment is even more challenging when a second large outbreak closely follows one that has depleted the susceptible pool. The ensemble filters and the basic PF (with sufficient particles) are more capable of adapting to such transitions and generating multiple peaks. In contrast, the MIF and pMCMC are not able to capture such variations. In the future, for seasons with multiple co-circulating strains, modeling these strains individually may improve the performance of the filters, especially the MIF and pMCMC.

In addition, the underlying assumptions of each filter can affect its performance. Among the ensemble filters, the EnKF appears to perform better than the EAKF (smaller RMS error, compared over all runs, 1-sided t-test, *p* = 2.97×10^−8^). The EnKF we use here is a stochastic filter in which random perturbations are added to the observed ILI+; the EAKF is a purely deterministic filter and relies solely on the observed ILI+ and OEV. Lei et al. [32] compared stochastic versus deterministic EnKFs under non-Gaussian systems, and found that the stochastic EnKF was more stable than the deterministic one in certain circumstances, such as in the presence of outliers. Our system is non-Gaussian, and outliers are common. Consistent with the findings of Lei et al. [32], the random perturbation added to the observed state variable seems to partially correct for these outliers and yield better performance.

Our SIRS model has only 2 variables and 4 parameters. For such a low-dimensional system, particle filter methods are numerically tractable and thus likely superior to the ensemble Kalman filter techniques, which include additional approximations. Indeed, the basic PF outperformed the other filters in some cases (Figure 3). Likewise, the RHF appeared to perform better than other filters in these cases; this filter, which has fewer constraints in the distribution of its posterior, seems to capture better abrupt changes in the dynamics of epidemics.

### Comparison of filters in forecasting

For the forecast of influenza, previous studies [4], [6], [7] indicate that the observation data type (e.g., ILI vs. ILI+), geographic and demographic characteristics (e.g., municipal area and population density), model form (e.g., whether daily humidity data are used), and the choice of filter method may all affect the accuracy of a forecast. In this study, we have focused exclusively on the choice of filter method. We found the filters that best simulated observed ILI+ time series did not necessarily generate better forecasts.

For peak timing forecast, filter accuracy varied temporally with certain filters excelling over specific windows of time but working less optimally during other periods. Overall, the particle filters produced more accurate forecasts before the observed peak, while the ensemble filters were more accurate after the actual peak. In addition, the performance of the model-filter frameworks varied geographically. We found that some cities tended to be more challenging for modeling (Figure 2) or forecasting (Figure S5); however, the cities most problematic for retrospective modeling (e.g., the 2 Oklahoma cities and the 5 Arizona cities) were not consistently the ones most challenging to forecast. Preliminary analysis of real-time forecasts made during the 2012–13 season in the U.S. also revealed a tendency toward greater forecast accuracy for cities with smaller populations, lower population density, and smaller geographic area [7]. As in He et al. [24], we found that parameter estimates tend to be biased for certain challenging outbreaks (e.g., overestimation of the infectious period, *D*, as shown in Figure S2); yet interestingly, these potentially biased estimates did not seem to degrade prediction over short time scales. Further examination of the effects of filter type on forecast accuracy at the municipal level, as well as factors modulating that accuracy, is warranted.

In addition, the forecast comparison presented here focused exclusively on the prediction of outbreak peak timing; filter performance may differ when forecasting other metrics, such as outbreak magnitude and duration. In particular, all six filters seem to overestimate outbreak magnitude when the forecast is made at a time close to the observed peak (Figure 5); this issue may arise from model misspecification or inadequate estimation of system parameters and initial conditions prior to forecast—issues that relate to model error and data richness. Furthermore, the characteristics, timing or location of an outbreak (e.g., unimodal vs. multimodal outbreaks, before vs. after an observed peak, small city vs. large city), may all affect filter performance. Further study is needed to determine the factors that make an outbreak more or less predictable by a given model-filter combination.

We have shown that for all the six filters, the confidence in a forecast increases as a larger percentage of individual forecasts (i.e. particles or ensemble members) agree with the mode predicted peak. This relationship, which essentially places greater confidence in predictions with less disagreement among particles or ensembles members, allows a forecaster to gauge the expected accuracy of a forecast in real time. Future study will look for similar relationships in the forecasts of other metrics such as influenza incidence at the peak.

## Supporting Information

Figure S1 **Effect of the number of particles/ensemble members.** The filters were run with either 300, 3000, or 10,000 particles/ensemble members, to model the historical ILI+ time series from 2003–04 to 2011–12 (excluding the pandemic seasons), for Atlanta, Boston, Chicago, Los Angeles, New York City, and Seattle. Each ILI+ time series was modeled using each filter 5 times, and Root Mean Squared (RMS) error was calculated for each run. Boxplots of the RMS errors over the 5 runs show the performance of each filter with different particle/ensemble sizes.
(PDF)

Figure S2 **Model parameter estimates for five AZ cities during the 2010–11 season.** Each panel (A–G) shows the time series of a model state (*S*, *I*, etc.) simulated by each of the six filters (specified in each plot) for the five AZ cities (specified on the top of each row). The ILI+ time series (A) are the same as shown in Figure 3 in the main text. Each filter was run 5 times; each colored line represents one run.
(PDF)

Figure S3 **Correlations for fitting historical ILI+ time series.** Each model-filter framework was run 5 times; the correlation between the predicted and observed ILI+ time series was calculated for each run; the color of each rectangle, corresponding to each city (y-axis) by each model-filter framework (x-axis), indicates the average correlation over the 5 repeated runs for epidemic seasons (A) 2003–04, (B) 2004–05, (C) 2005–06, (D) 2006–07, (E) 2007–08, (F) 2010–2011, and (G) 2011–12.
(TIF)

Figure S4 **Accuracy vs. ensemble spread.** All forecasts, 565,500 in total, were first categorized according to mode predicted peak, e.g., 1–3 weeks in the future (the first row) or 3–5 weeks in the past (the last row); within each category, forecasts were further grouped by the range of log ensemble variance, as indicated on the x-axis; the accuracy of forecasts within each bin were then calculated, as shown on the y-axis. Dot size indicates the portion of forecasts within each bin. Each column (A–F) shows the relationship between the forecast accuracy and the logarithm ensemble variance for a different filter.
(TIF)

Figure S5 **Mode predicted peak accuracy as a function of time relative to the observed peak.** Accuracy for each filter is averaged over all seasons and all runs for each city (i.e. 7 seasons and 5 runs), for forecasts made 3 wk before (A), 1 wk before (B), at (C), 1 wk after (D), or 3 wk after (E) the local peak outbreak of the corresponding season.
(TIF)

Text S1 **Supporting information.** Supplemental methods, testing, and discussion.
(DOC)
