# Supplementary figures and images for: Comparison of Filtering Methods for the Modeling and Retrospective Forecasting of Influenza Epidemics
Source: PLoS Comput Biol. 2014 Apr 24;10(4):e1003583. doi: 10.1371/journal.pcbi.1003583 (PMC3998879; doi:10.1371/journal.pcbi.1003583)

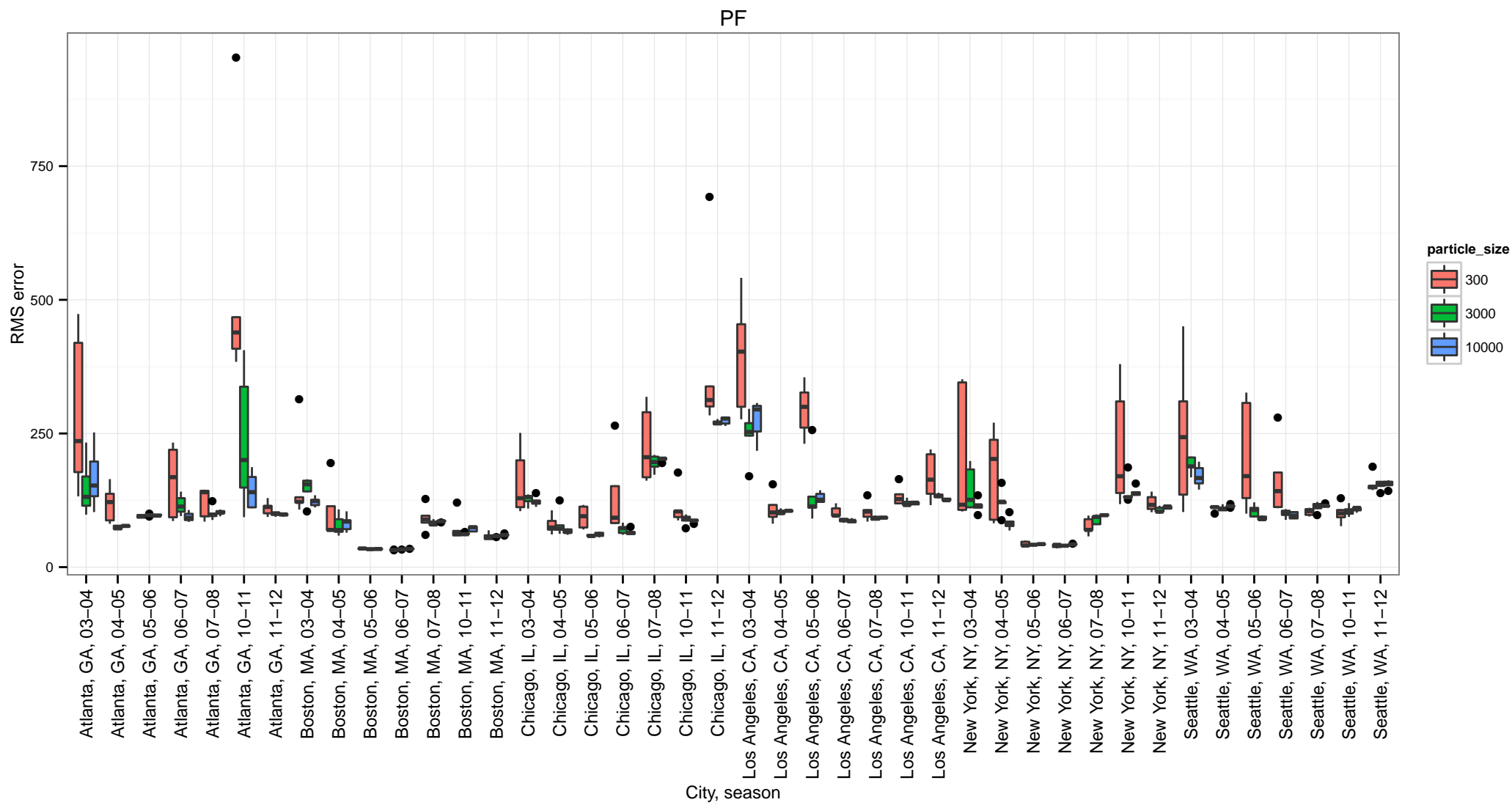

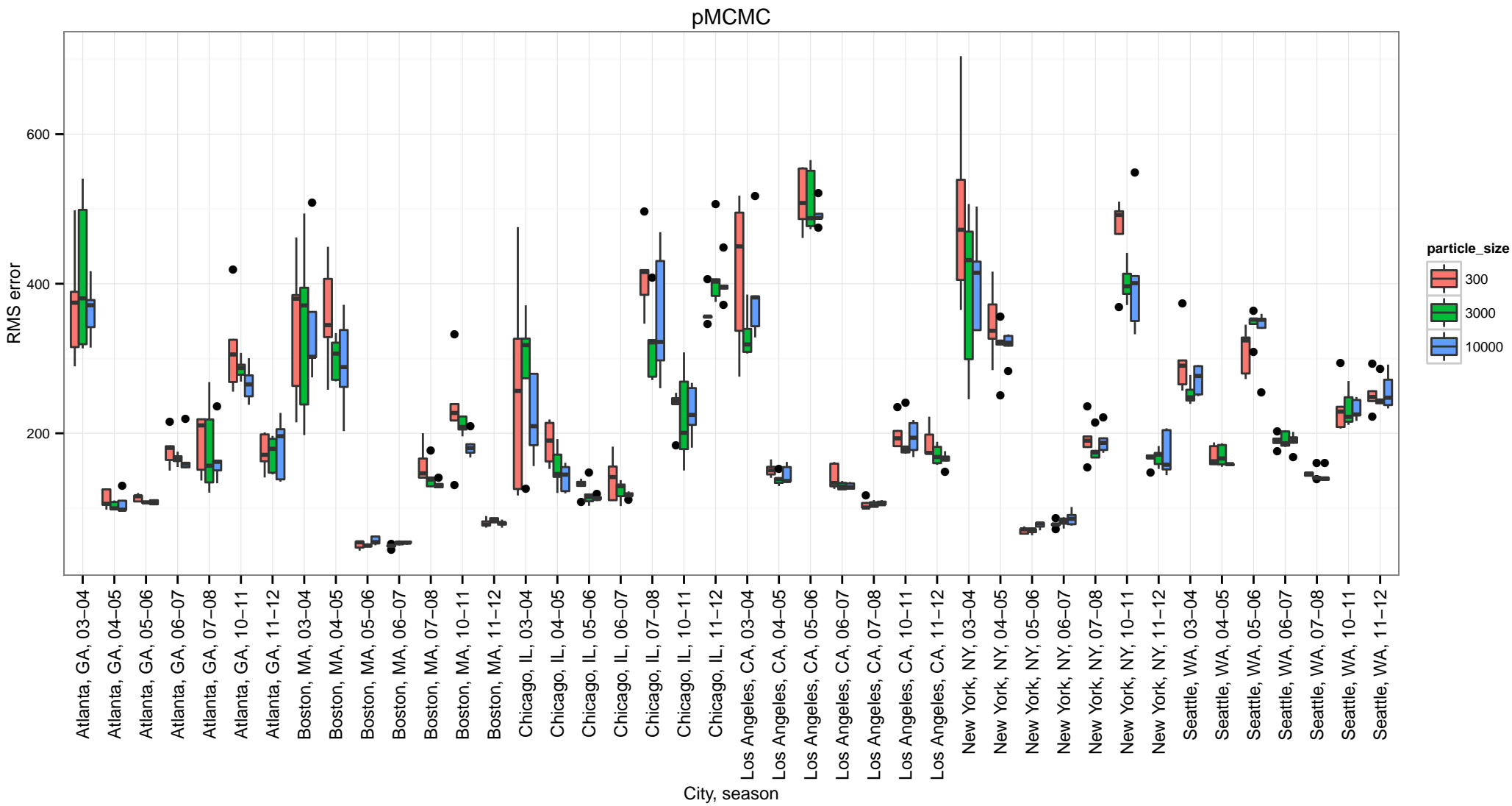

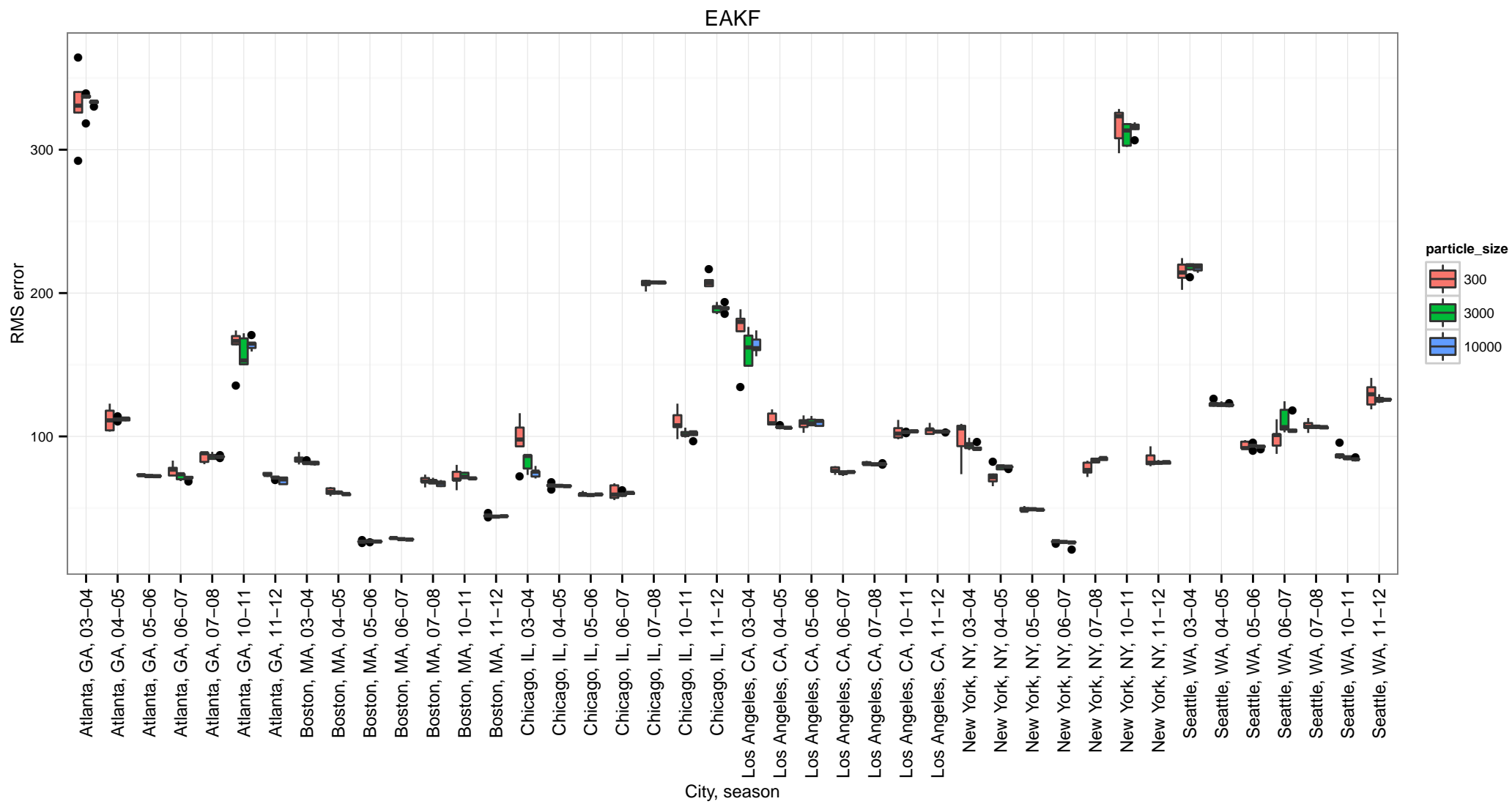

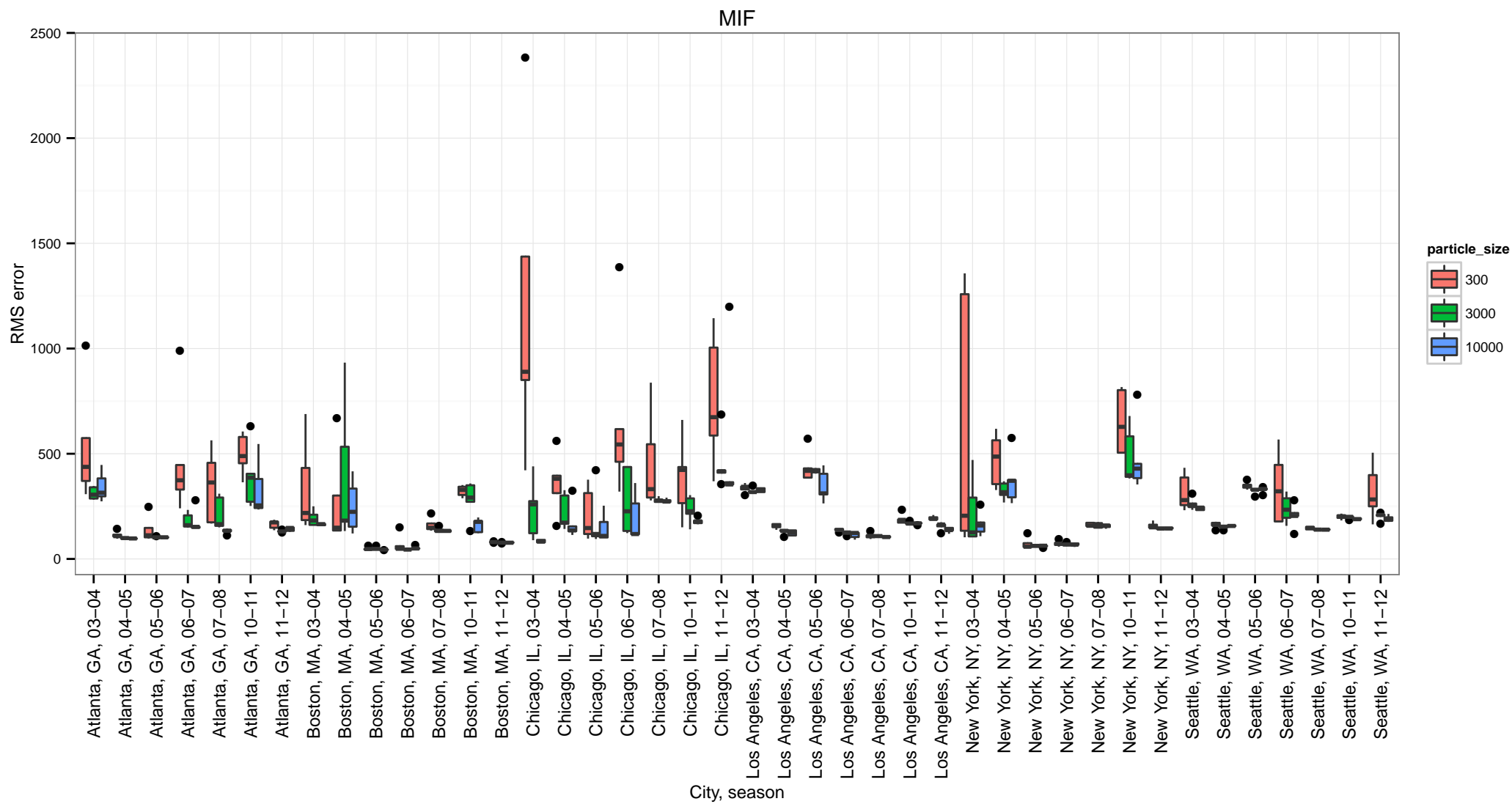

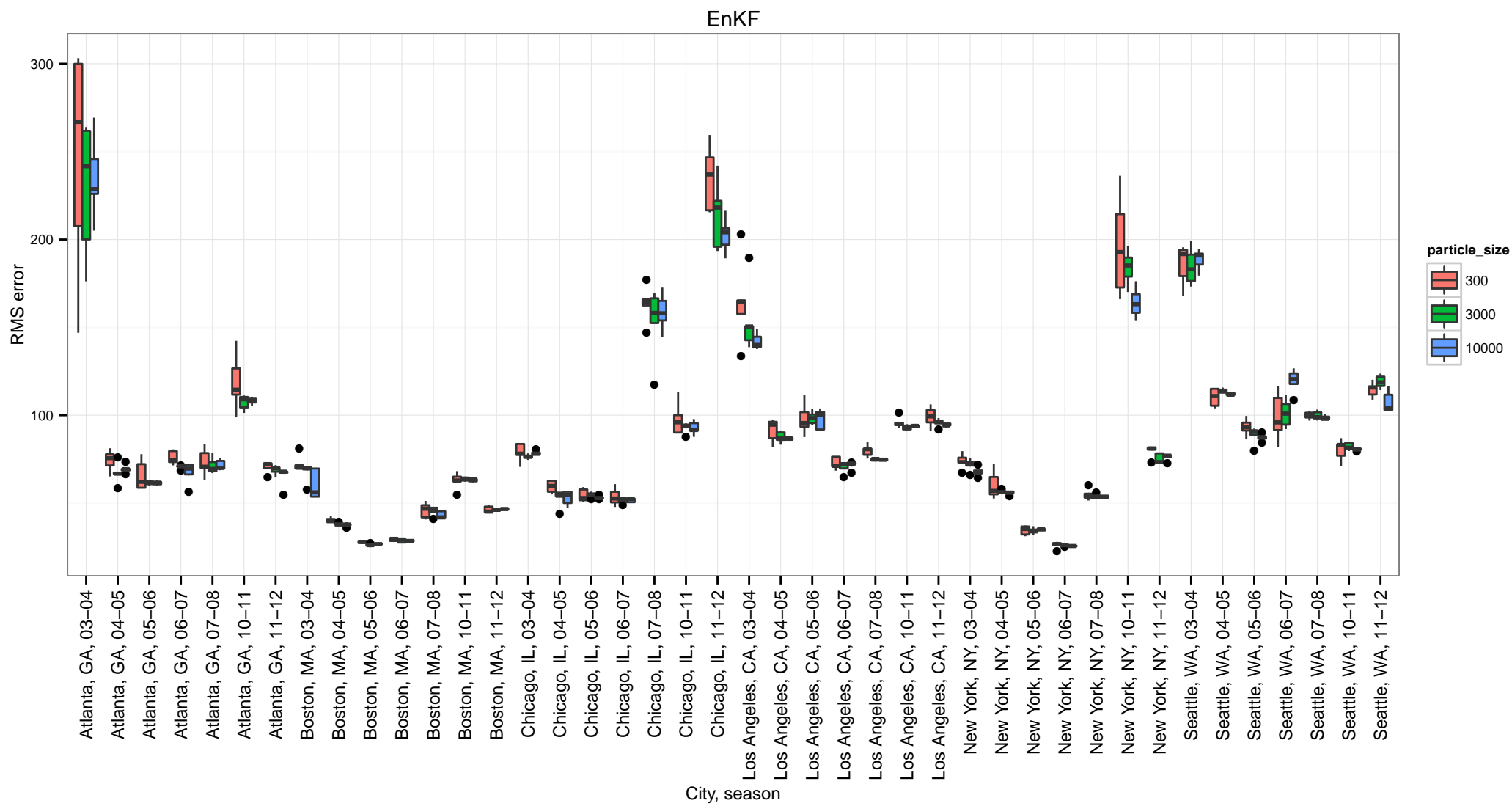

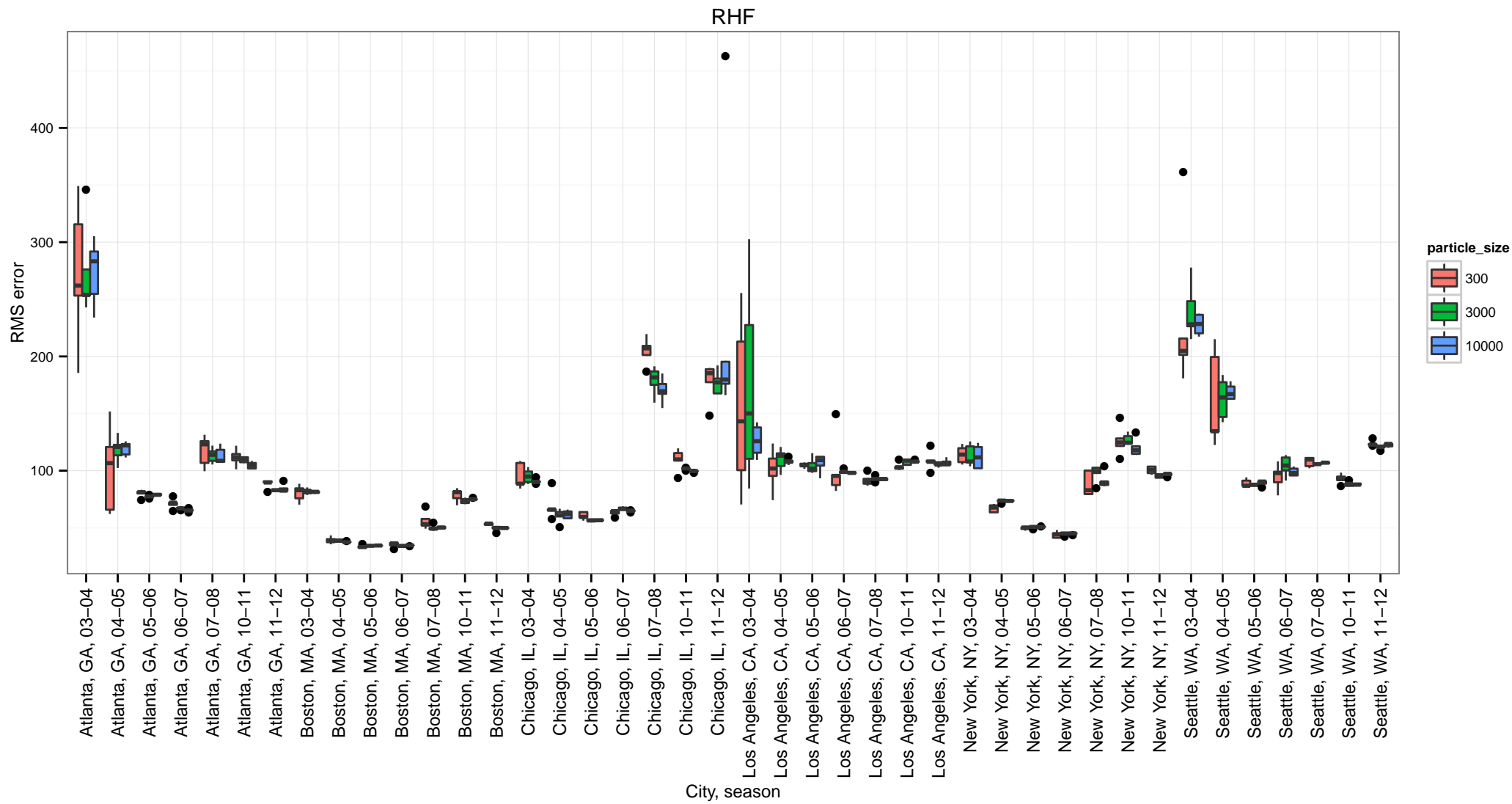

Supplement: Figure S1 — Effect of the number of particles/ensemble members. The filters were run with either 300, 3000, or 10,000 particles/ensemble members, to model the historical ILI+ time series from 2003–04 to 2011–12 (excluding the pandemic seasons), for Atlanta, Boston, Chicago, Los Angeles, New York City, and Seattle. Each ILI+ time series was modeled using each filter 5 times, and Root Mean Squared (RMS) error was calculated for each run. Boxplots of the RMS errors over the 5 runs show the performance of each filter with different particle/ensemble sizes. (PDF) [file pcbi.1003583.s001.pdf]

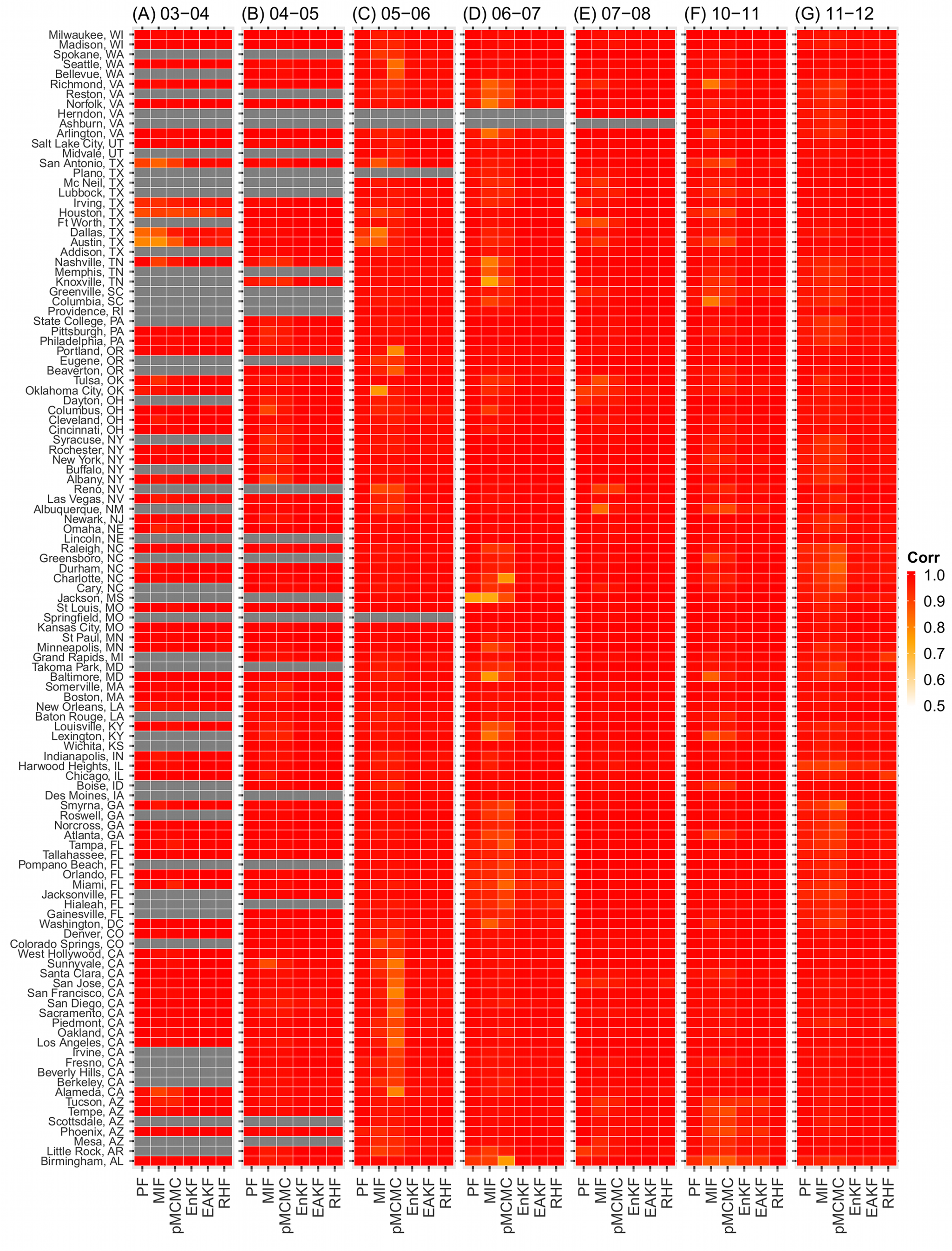

Supplement: Figure S3 — Correlations for fitting historical ILI+ time series. Each model-filter framework was run 5 times; the correlation between the predicted and observed ILI+ time series was calculated for each run; the color of each rectangle, corresponding to each city (y-axis) by each model-filter framework (x-axis), indicates the average correlation over the 5 repeated runs for epidemic seasons (A) 2003–04, (B) 2004–05, (C) 2005–06, (D) 2006–07, (E) 2007–08, (F) 2010–2011, and (G) 2011–12. (TIF) [file pcbi.1003583.s003.tif]

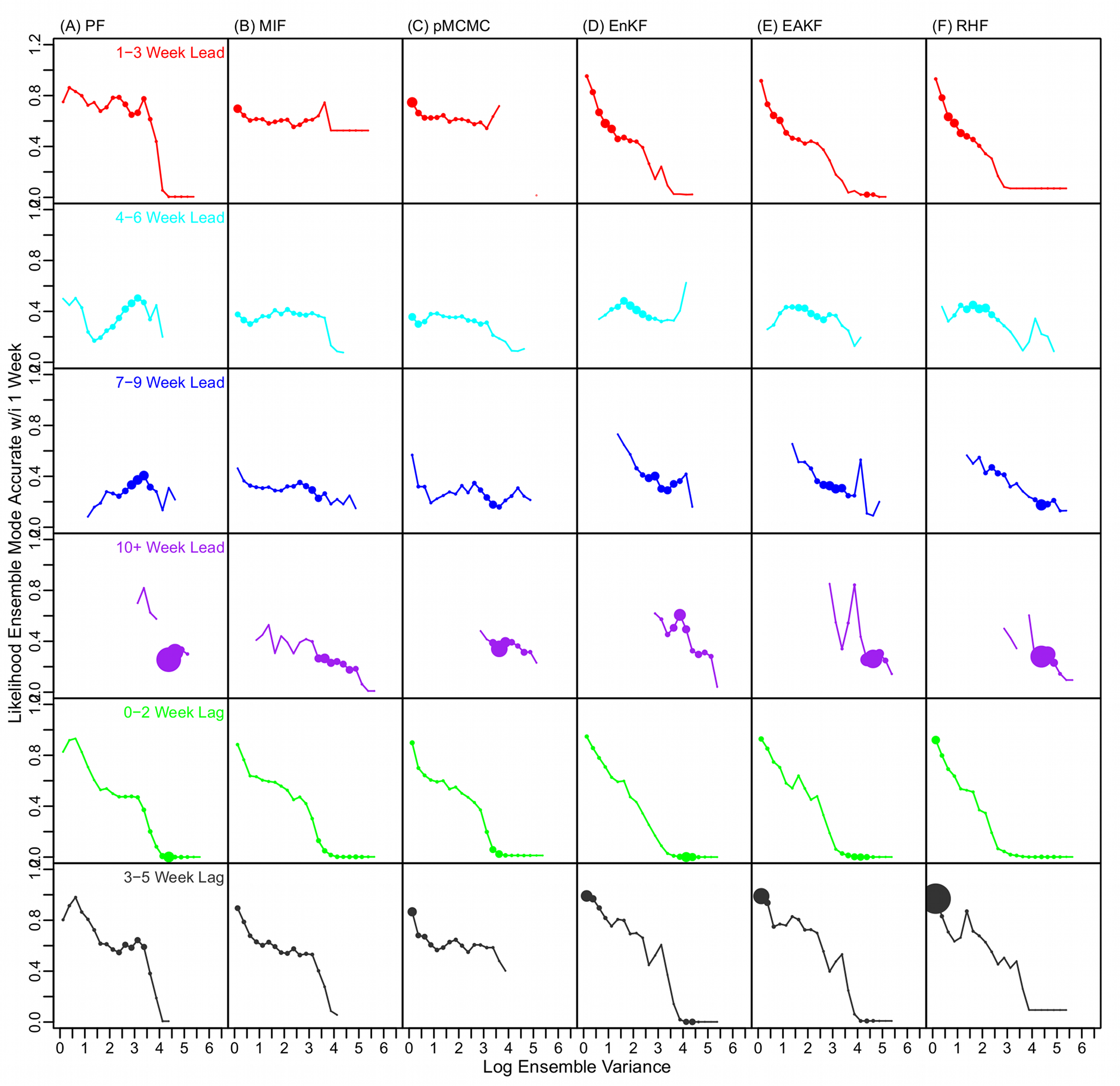

Supplement: Figure S4 — Accuracy vs. ensemble spread. All forecasts, 565,500 in total, were first categorized according to mode predicted peak, e.g., 1–3 weeks in the future (the first row) or 3–5 weeks in the past (the last row); within each category, forecasts were further grouped by the range of log ensemble variance, as indicated on the x-axis; the accuracy of forecasts within each bin were then calculated, as shown on the y-axis. Dot size indicates the portion of forecasts within each bin. Each column (A–F) shows the relationship between the forecast accuracy and the logarithm ensemble variance for a different filter. (TIF) [file pcbi.1003583.s004.tif]

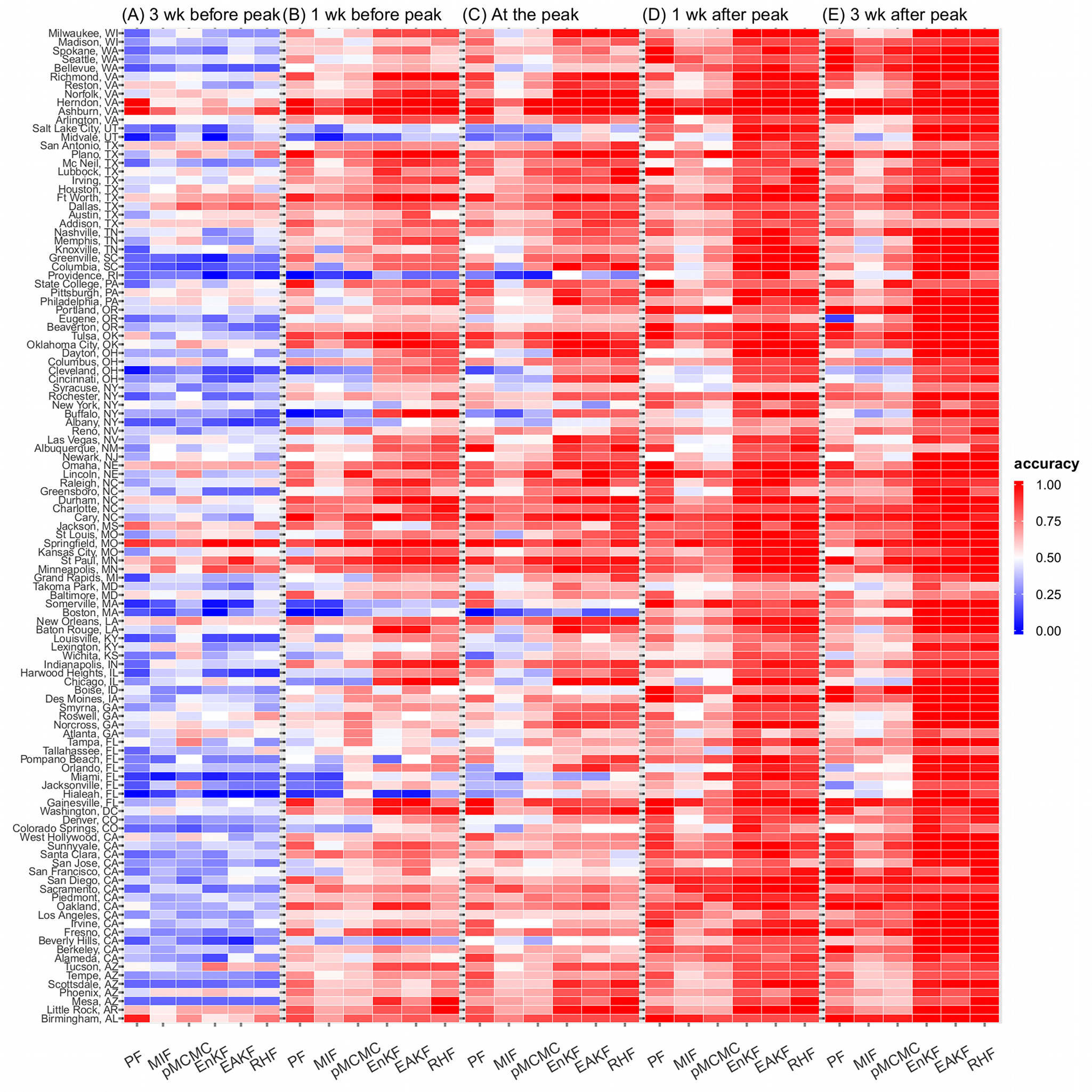

Supplement: Figure S5 — Mode predicted peak accuracy as a function of time relative to the observed peak. Accuracy for each filter is averaged over all seasons and all runs for each city (i.e. 7 seasons and 5 runs), for forecasts made 3 wk before (A), 1 wk before (B), at (C), 1 wk after (D), or 3 wk after (E) the local peak outbreak of the corresponding season. (TIF) [file pcbi.1003583.s005.tif]
