# Supplementary material for: Comparison of Filtering Methods for the Modeling and Retrospective Forecasting of Influenza Epidemics
Source: PLoS Comput Biol. 2014 Apr 24;10(4):e1003583. doi: 10.1371/journal.pcbi.1003583 (PMC3998879; doi:10.1371/journal.pcbi.1003583)
Supplement: Figure S2 — Model parameter estimates for five AZ cities during the 2010–11 season. Each panel (A–G) shows the time series of a model state (S, I, etc.) simulated by each of the six filters (specified in each plot) for the five AZ cities (specified on the top of each row). The ILI+ time series (A) are the same as shown in Figure 3 in the main text. Each filter was run 5 times; each colored line represents one run. (PDF) [file pcbi.1003583.s002.pdf]

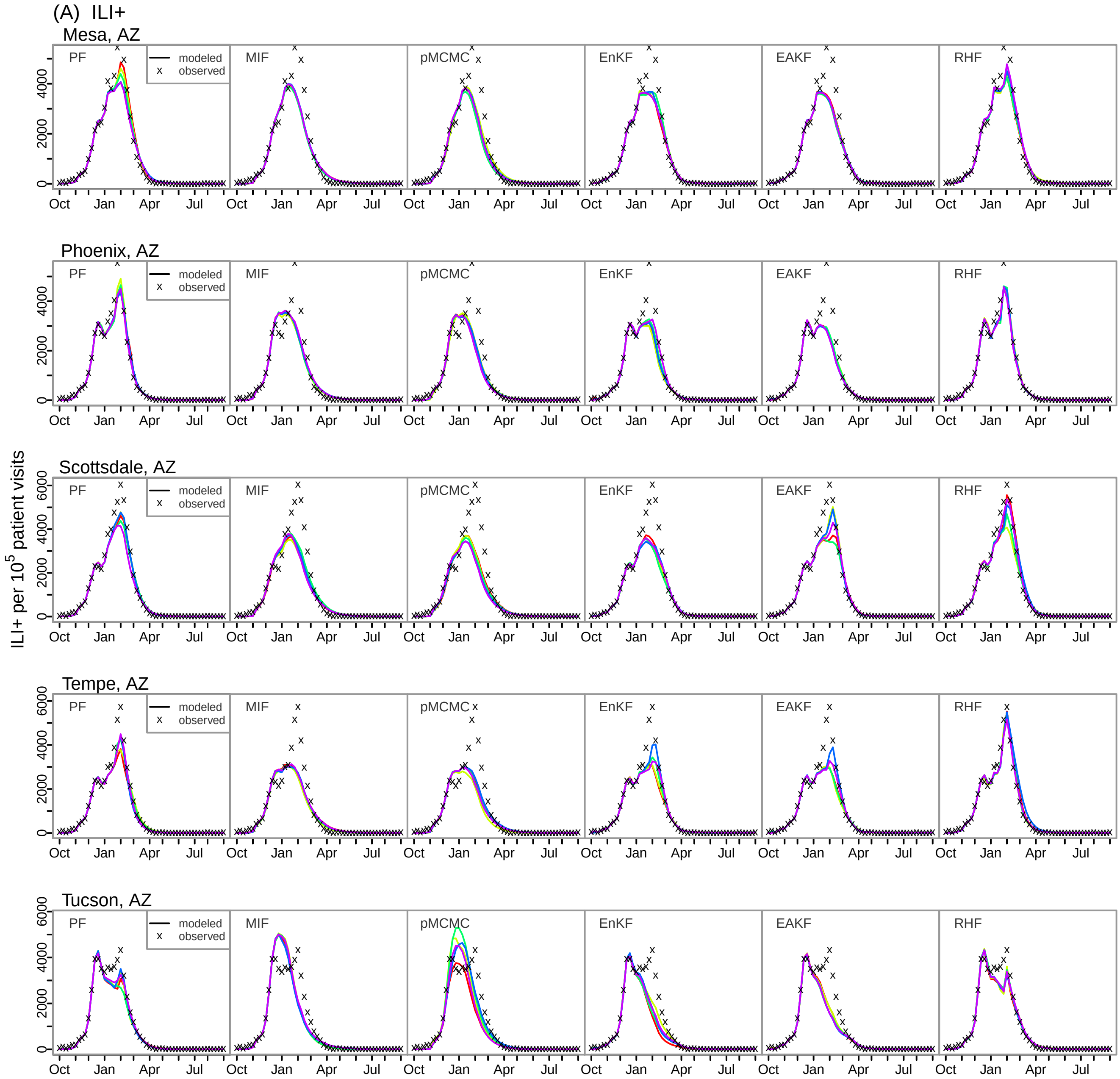

(A) ILI+
Mesa, AZ
Phoenix, AZ
Scottsdale, AZ
Tempe, AZ
Tucson, AZ
PF
MIF
pMCMC
EnKF
EAKF
RHF
modeled
observed
ILI+ per 10^5 patient visits
0
2000
4000
6000
Oct
Jan
Apr
Jul

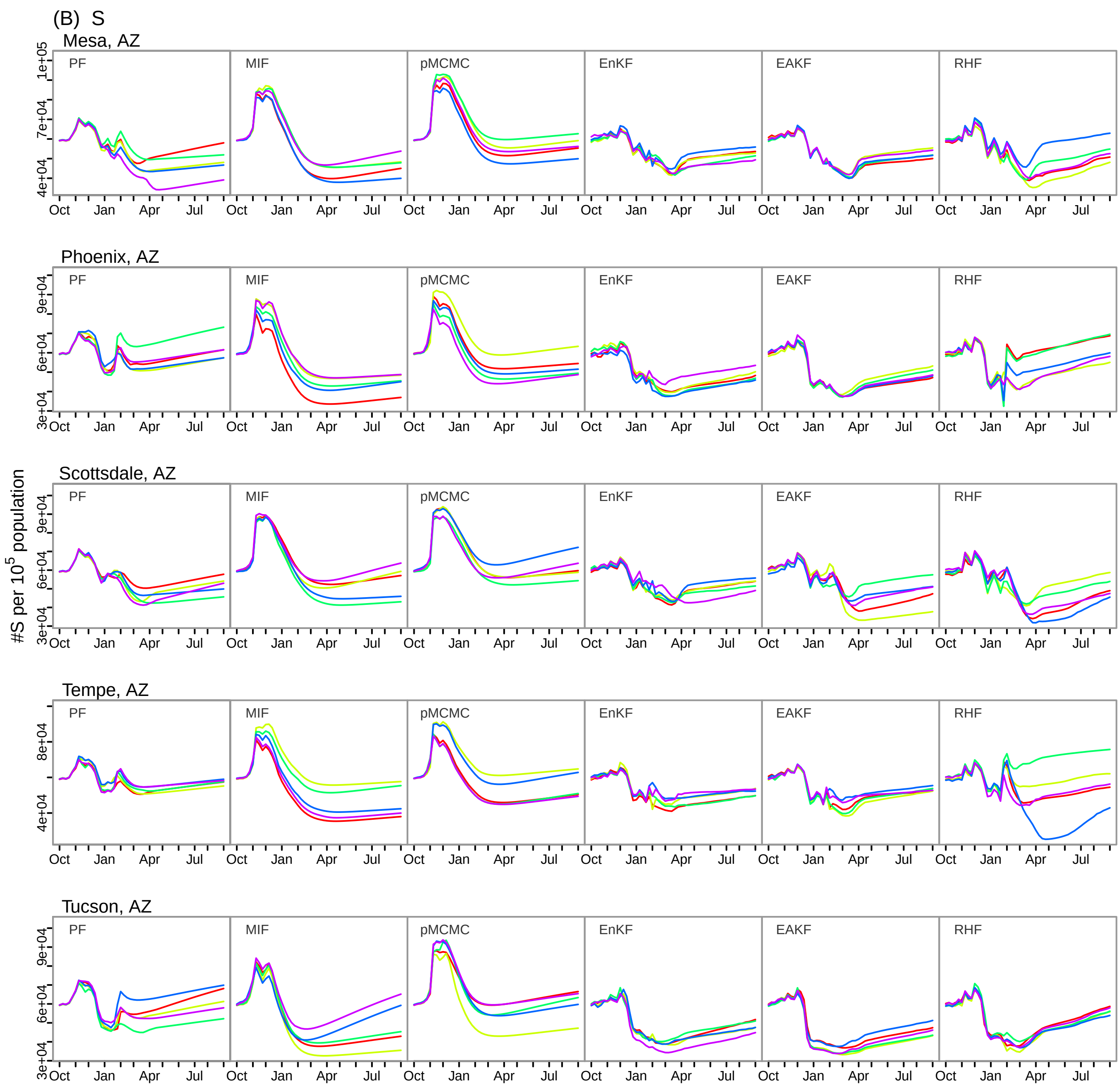
(B) S
Mesa, AZ
Phoenix, AZ
Scottsdale, AZ
Tempe, AZ
Tucson, AZ
PF
MIF
pMCMC
EnKF
EAKF
RHF
Oct
Jan
Apr
Jul
#S per 10^5 population
1e+05
7e+04
4e+04
9e+04
6e+04
3e+04
8e+04

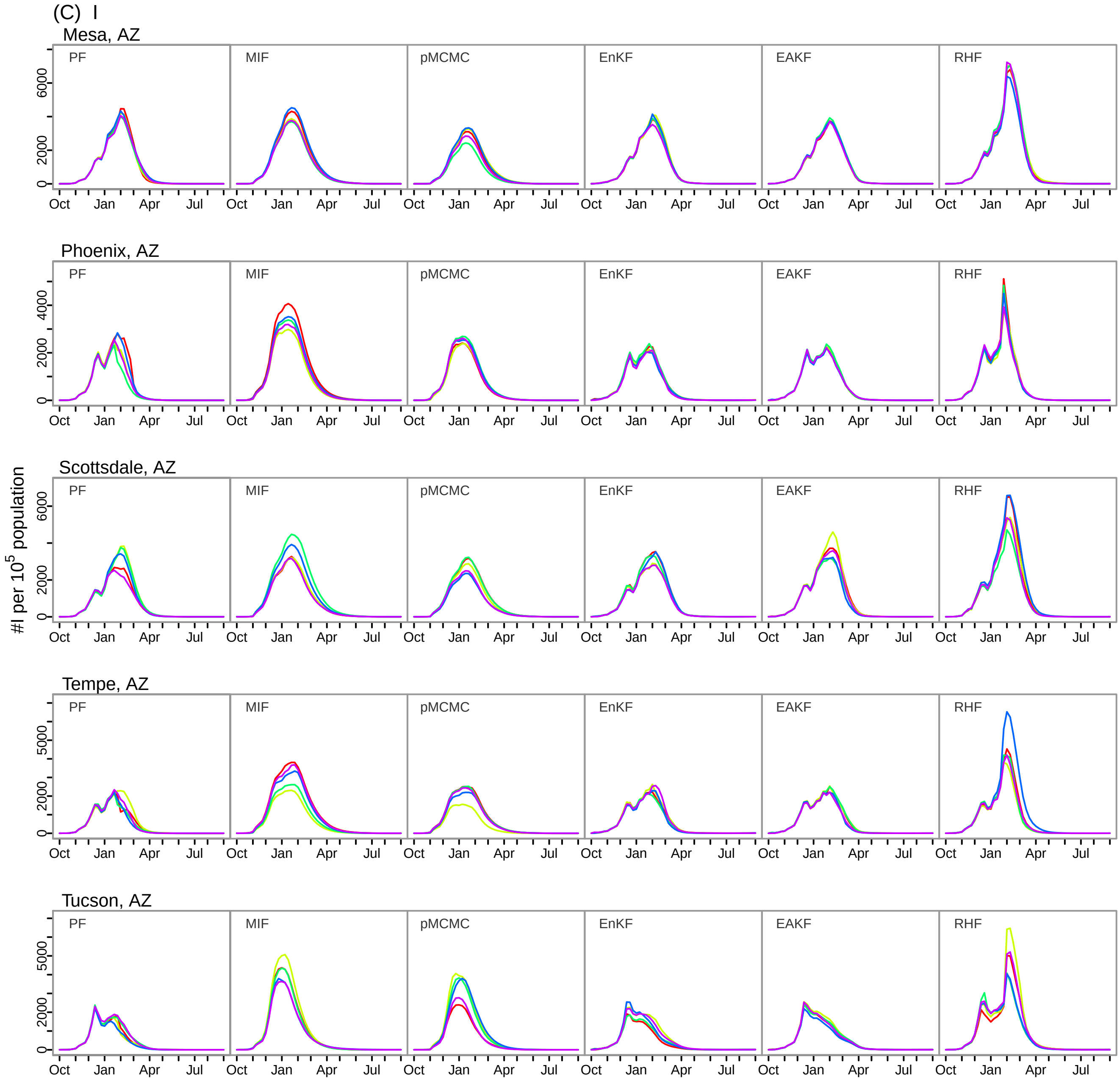
(C) I
#I per 10^5 population
Mesa, AZ
Phoenix, AZ
Scottsdale, AZ
Tempe, AZ
Tucson, AZ
PF
MIF
pMCMC
EnKF
EAKF
RHF
Oct
Jan
Apr
Jul
0
2000
4000
5000
6000

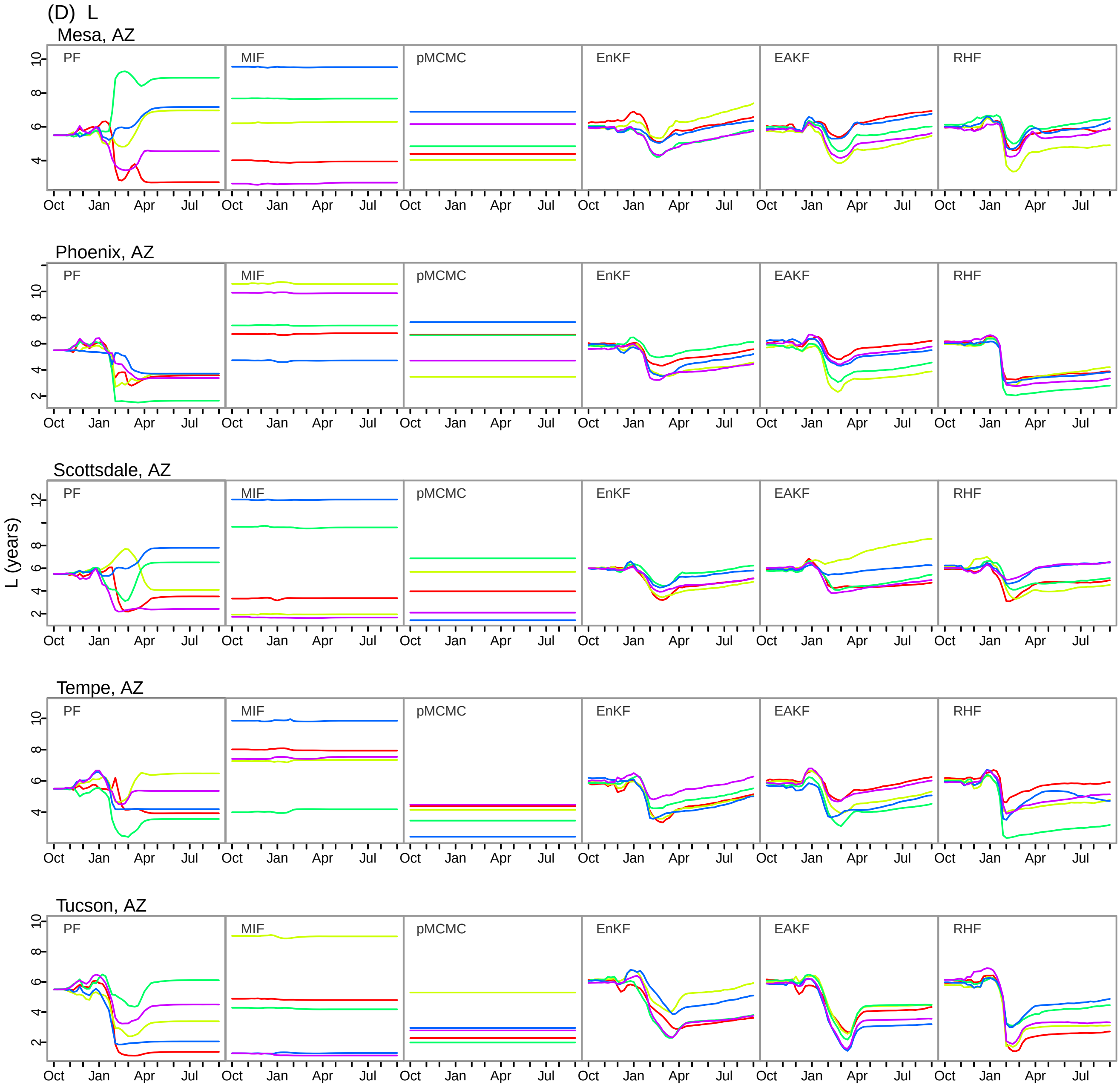

(D) L
Mesa, AZ
Phoenix, AZ
Scottsdale, AZ
Tempe, AZ
Tucson, AZ
PF
MIF
pMCMC
EnKF
EAKF
RHF
L (years)
Oct
Jan
Apr
Jul

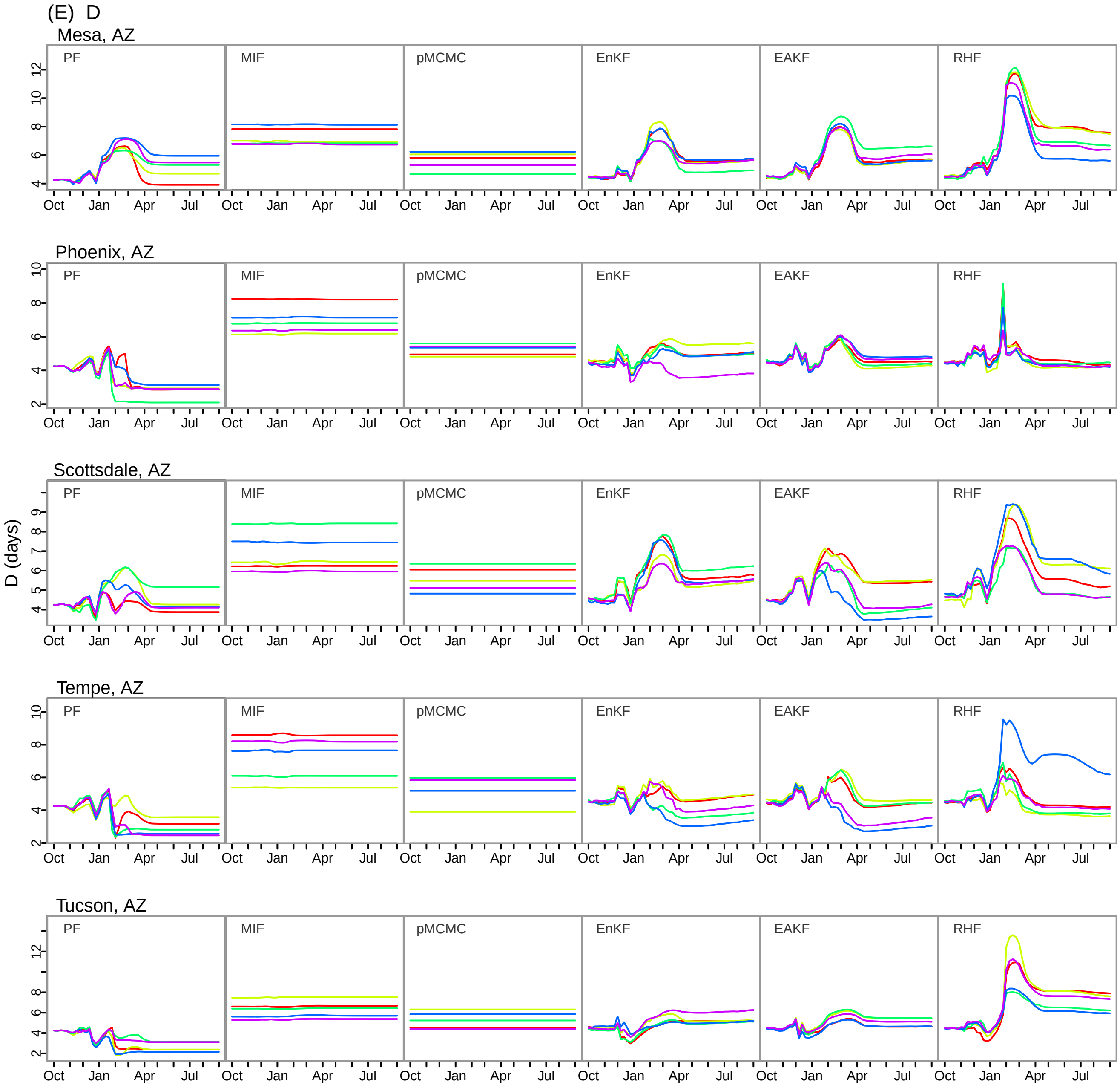
(E) D
Mesa, AZ
Phoenix, AZ
Scottsdale, AZ
Tempe, AZ
Tucson, AZ
PF
MIF
pMCMC
EnKF
EAKF
RHF
D (days)
Oct
Jan
Apr
Jul

(F) R0max

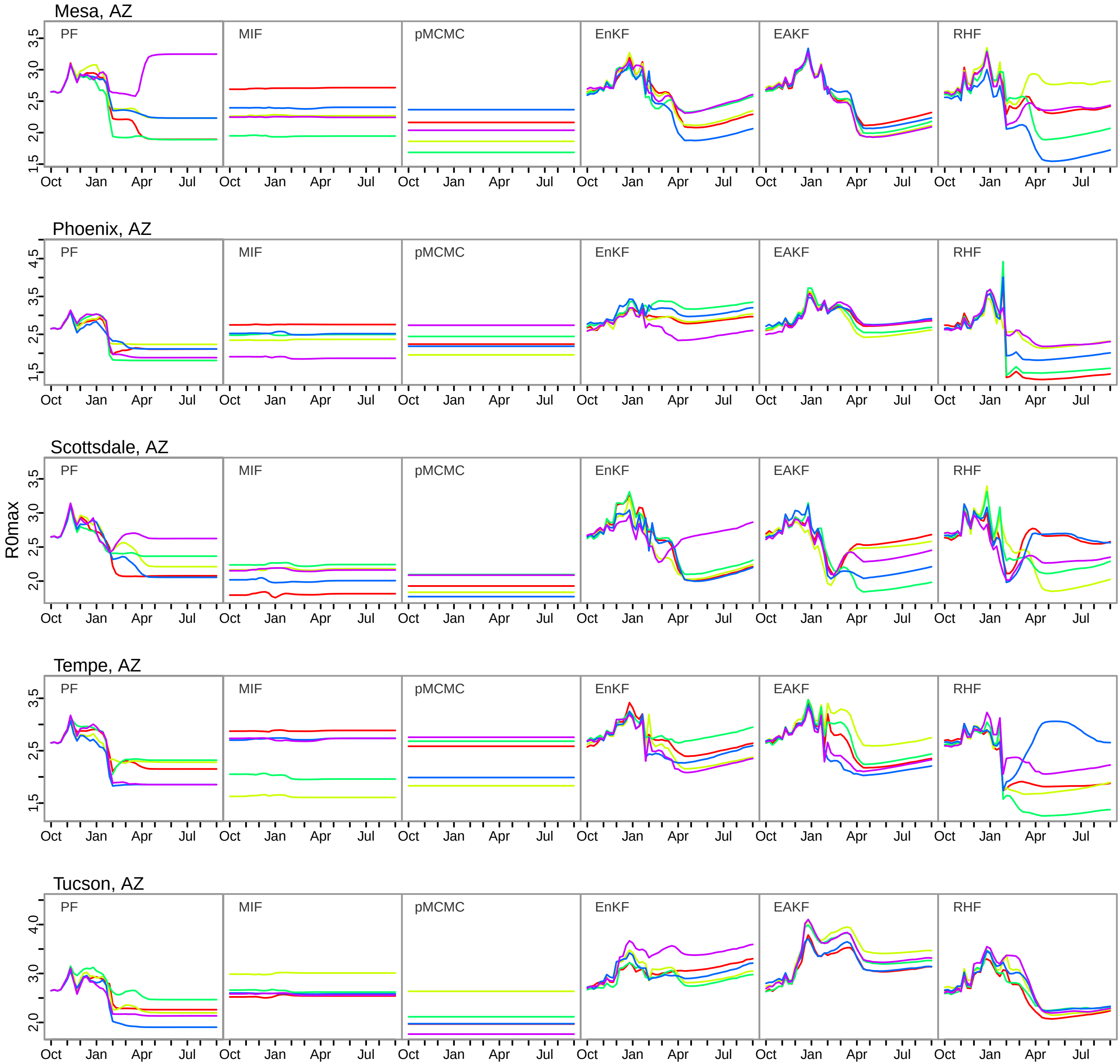
Mesa, AZ
Phoenix, AZ
Scottsdale, AZ
Tempe, AZ
Tucson, AZ
PF
MIF
pMCMC
EnKF
EAKF
RHF
R0max
1.5
2.0
2.5
3.0
3.5
4.0
4.5
Oct
Jan
Apr
Jul

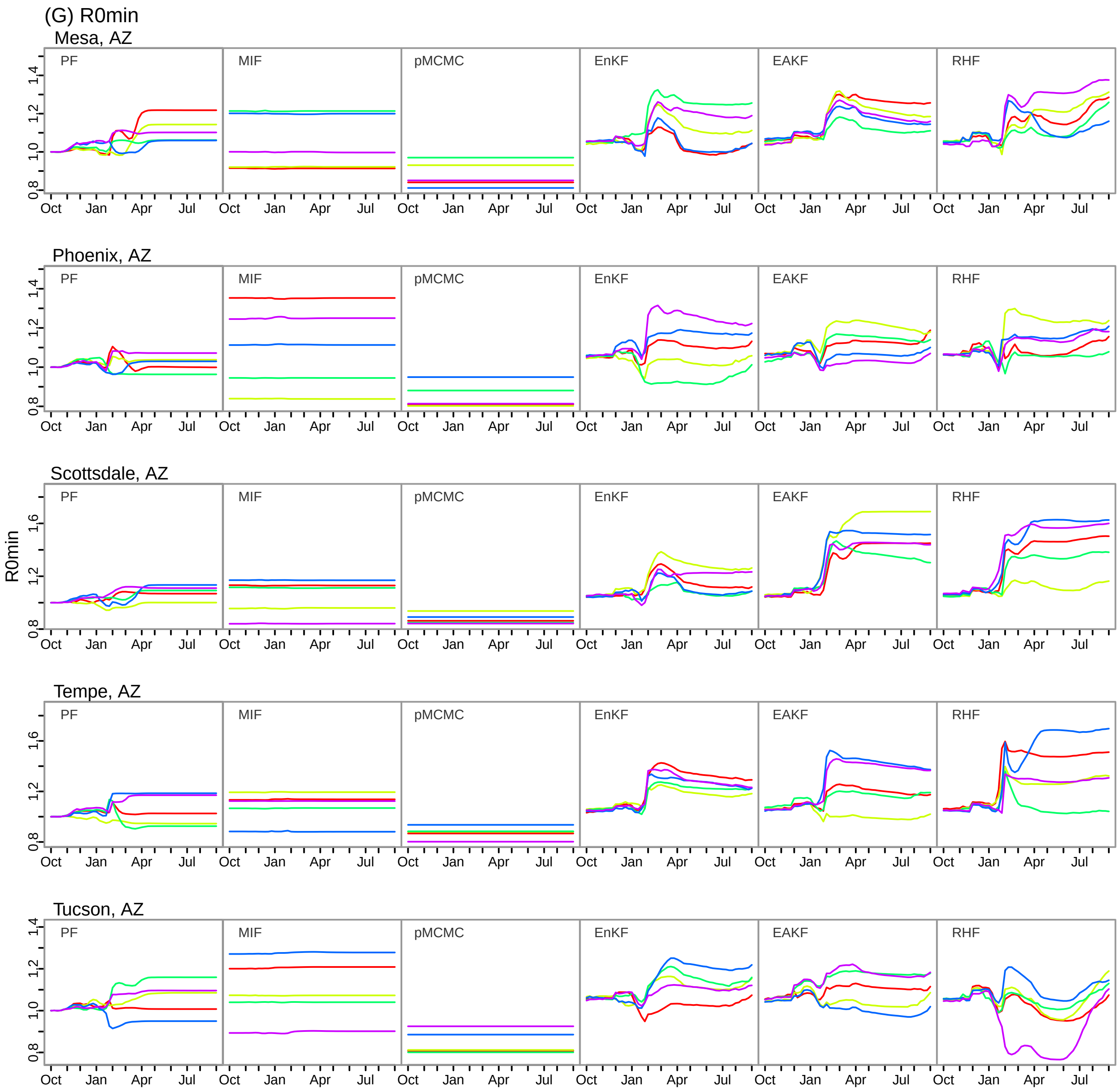
(G) R0min
Mesa, AZ
Phoenix, AZ
Scottsdale, AZ
Tempe, AZ
Tucson, AZ
PF
MIF
pMCMC
EnKF
EAKF
RHF
R0min
Oct
Jan
Apr
Jul
