# Supplementary material for: Comparison of Filtering Methods for the Modeling and Retrospective Forecasting of Influenza Epidemics
Source: PLoS Comput Biol. 2014 Apr 24;10(4):e1003583. doi: 10.1371/journal.pcbi.1003583 (PMC3998879; doi:10.1371/journal.pcbi.1003583)
Supplement: Text S1 — Supporting information. Supplemental methods, testing, and discussion. (DOC) [file pcbi.1003583.s006.doc]

**Text S1**

**Comparison of filtering methods for the modeling and retrospective forecasting of influenza epidemics**

Wan Yang, Alicia Karspeck, Jeffrey Shaman

**Specific Humidity Data**

Absolute humidity (AH), represented by specific humidity (SH), is included in our SIRS model (as described below) as a seasonal driving factor for influenza epidemics . SH data were compiled from the National Land Data Assimilation System (NLDAS) project-2 dataset. These data are derived through spatial interpolation, temporal disaggregation and vertical adjustment from station measurements and National Center for Environmental Prediction North American Regional Reanalysis . The gridded NLDAS meteorological data are available in hourly time steps on a 0.125° regular grid from 1979 through the present . Local SH data for each of the 115 cities included in these forecasts were assembled for 1979-2011. These hourly data were then averaged to daily resolution. A 1979-2002 (24 year) daily climatology was then constructed for each city and used as the daily specific humidity forcing for all retrospective forecasts.

**Implementation of each filter**

(1) PF

The PF used in this study is primarily based on Algorithm 3 in . The PF consists of a prediction and an update, done alternatively and repeatedly until all available observations have been assimilated. Briefly, an initial set of particles (10,000 in this study, and same for the MIF and pMCMC), each including the state variables (i.e., number of susceptible, *S*, and infectious, *I*) and 4 parameters (i.e., the immunity period, *L*, infectious period, *D*, and daily maximum and minimum reproductive numbers, *R_0max_* and *R_0min_*) are generated from a Latin hypercube function widely covering the state space. Particles are propagated according to SIRS model equations , which generate a prediction, i.e., the prior distribution. Whenever a new observation becomes available, the particles are weighted according to the likelihood, and updated to generate the posterior distribution.

Resampling is performed whenever the effective particle size drops below half of the total particle count (i.e. 10,000). To avoid particle impoverishment, a regularization step is incorporated in the resampling step.

(2) MIF

The MIF in this study was done mainly according to Ionides et al. . At the first iteration, particles are initialized randomly with broad variance. With each subsequent iteration, estimates from the previous iteration (e.g., the mean of each parameter estimate) are used to generate new particles for the current iteration, with the covariance of the parameters reduced exponentially. As the variance of the parameters approaches zero, the filter generates parameter estimates with maximum likelihood (see ). The parameter estimates of the final iteration (i.e. integration through the complete time series) vary little during that final integration. In this study, the state variables (i.e., *S* and *I*) were generated from a Latin hypercube function widely covering the state space, while the parameters were generated using a multinormal distribution. We used 30 iterations and 0.95 for the discount factor (α, same as in ). The filtering step in the MIF was performed using the same algorithm as for the basic PF, described above; however, regularization was only applied to the state variables.

(3) pMCMC

The pMCMC used in this study is based on the particle marginal Metropolis-Hastings sampler in . It applies the MCMC method to explore the state space of model parameters and the particle filtering technique to compute the likelihood over time. Each particle thus only includes the state variables (i.e., *S* and *I*); and all particles share the same set of model parameters proposed by the MCMC step. Acceptance of parameter proposal is based on the joint likelihood over all observations per the Metropolis-Hastings algorithm.

A successful MCMC relies on the capability of the algorithm to generate proposals, i.e., values for the parameters to be estimated, which can efficiently explore the state space. A random walk, with thousands to millions of MCMC steps, is the norm in MCMC algorithms. However, for inference of a long time series, it is impractical to have millions or even thousands of MCMC steps, due to the computational expense. It is thus challenging to propose limited sets of model parameters that can cover a multi-dimensional state space. To cover the state space as broadly as possible and avoid the MCMC being trapped in local maxima, we generated parameter proposals using a Latin hypercube function. To search for an optimal parameter set, we conducted the pMCMC with two stages; in the first, proposals are generated with broader boundaries, in the second, proposals are generated centered at the best proposal yielded from the first stage but within a smaller state space. A total of 100 MCMC steps (i.e., 100 proposals) were used, with 80 for the first stage. This strategy resulted in a proposal acceptance rate of ~10%. The filtering step in pMCMC uses an auxiliary filter as adopted in ; however, we modified the algorithm to resample only when the effective particle count dropped by half, and applied regularization to the state variables during resampling.

(4) EnKF

Like the particle filters, the ensemble filters we use here consist of repeated cycles of short-term prediction (to generate priors) followed by an update triggered by an incoming observation (generating a posterior). The EnKF algorithm was performed according to . Briefly, an initial ensemble is generated then integrated forward until new datum is received, upon which this new datum is assimilated into the model. This is done sequentially through time. For each ensemble member, the prior, or prediction, is made according to the SIRS model, and the posterior of the observed variable is calculated as a weighted average of its prior and the measurement according to Eq.7 of the main text.

To perform the EnKF, we added Gaussian white noise to the measurement data to mimic multiple measurements. We used 300 ensemble members for each run, for all ensemble filters. To avoid filter divergence, we included a simple multiplicative covariance inflation step, which increased the spread of ensemble prior at each step before the update. An inflation factor (λ) of 1.03 was adopted for all ensemble filters.

(5) EAKF

The EAKF algorithm, based on , is a deterministic variant of the EnKF. It adjusts the ensemble members such that the posterior mean and variance are equivalent to what is predicted by Bayes theorem, assuming that the prior and likelihood are Gaussian. Specifically, the posterior of the observed variable is calculated as:

| $x_{k,post}^{n}=\left(\frac{\sigma_{k,obs}^{2}}{\sigma_{k,obs}^{2}+\sigma_{k,prior}^{2}}\overline{x_{k,prior}}+\frac{\sigma_{k,prior}^{2}}{\sigma_{k,obs}^{2}+\sigma_{k,prior}^{2}}z_{k}\right)+\sqrt{\frac{\sigma_{k,obs}^{2}}{\sigma_{k,obs}^{2}+\sigma_{k,prior}^{2}}}\left(x_{k,prior}^{n}-\overline{x_{k,prior}}\right)$ | [S1] |
| --- | --- |

where, for week *k,* $x_{k,post}^{n}$ and $x_{k,prior}^{n}$ are the observed state variable posterior and prior, respectively, for the *n*th ensemble member, and $\overline{x_{k,prior}}$ is the observed state variable mean ensemble prior. Per Eq. S1, after the initialization of ensemble, the model is run fully deterministically.

(6) RHF

The RHF is based on . The ensemble members of the prior are ranked in ascending order and used to estimate a continuous prior distribution. A continuous likelihood function is then built by linking the point-wise likelihood estimates at each prior value. Finally, the posterior distribution is calculated by taking the product of the prior and the likelihood. The new position of each posterior ensemble member is where the cumulative posterior distribution is equal to n/(N+1), where n = 1, 2, …, N and N is the ensemble size. Specifically, in this study, the ensemble prior density is a histogram (i.e., a step function), and the likelihood density for the ensemble is line segments linking those point-wise likelihood estimates at each prior value. The posterior density is then also line segments with ends at the prior values. Therefore, posterior values are located by solving quadratic functions.

**Selecting particle/ensemble size for the six filters**

Particle filters approximate the distribution in continuous state space using discrete points (i.e., particles). To effectively sample this continuous state space, a large collection of particles is required. Additional approaches are usually applied to avoid particle impoverishment, especially for nonlinear dynamical systems. Common methods include conditional resampling based on the effective particle size, and regularization, which jitters the particles upon resampling as opposed to simply generating redundant particles . We applied both conditional resampling and regularization to all three particle filters. To test the optimal particle size, we ran the three particle filters with either 300, 3000, or 10000 particles, on historical ILI+ time series for Atlanta, Boston, Chicago, Los Angeles, New York, and Seattle, for the seven flu seasons. Increasing the particle size from 300 to 3000 significantly improved the performance (lower RMS error or higher correlation, pairwise paired t-test, 1 sided, *p*<0.001). Increasing the particle size to 10000 did not significantly improve their performance further; however, the variance in the outputs of different runs decreased with increasing particle size (Figure S1). To achieve higher consistency in filtering output, we therefore used 10000 particles for all the particle filters.

Ensemble filters, similar to the particle filters, employ a Monte Carlo based approach that generates an ensemble of N trajectories to sample the state space. For the EnFK and EAKF, the ensemble also provides an estimate of the joint distribution of state space (through the covariances of the observed variable and the unobserved variables, as shown in Eq. 8 in the main text). Therefore, the quality of ensemble filters also depends on the ensemble size. That being said, a much smaller number of ensemble members can typically be used for the same application. Still, the use of a finite number of ensembles can lead to systematic underestimation of the spread of the prior , which may cause ‘filter divergence’, in which the filter grows ‘over-confident’, gives less and less weight to the observations, and eventually diverges from the true state. To overcome filter divergence, one could apply a number of approaches, such as covariance localization, double-ensemble Kalman filter, and covariance inflation . In this study, we incorporated covariance inflation in all ensemble filters, which simply broadens the prior distribution to counter loss of variance over the filtering process . Our tests show that covariance inflation is critical in preventing the ensemble filters from divergence, especially when the ILI+ time series contain multiple peaks. With covariance inflation, the ensemble filters perform well with an ensemble size of 300, and did not significantly benefit from larger ensemble size (e.g., 3000 or 10000 ensemble members, pairwise paired t-test, 1 sided, *p*>0.05, and Figure S1).

All code for the six model-filter frameworks was written and run in R (http://www.R-project.org). The code for the six influenza forecast frameworks is available from our website: http://cpid.iri.columbia.edu/Refs.html.

**References**

1. Shaman J, Kohn M (2009) Absolute humidity modulates influenza survival, transmission, and seasonality. Proc Natl Acad Sci USA 106: 3243-3248.

2. Mesinger F, DiMego G, Kalnay E, Mitchell K, Shafran PC, et al. (2006) North American regional reanalysis. Bulletin of the American Meteorological Society 87: 343-360.

3. Cosgrove BA, Lohmann D, Mitchell KE, Houser PR, Wood EF, et al. (2003) Real-time and retrospective forcing in the North American Land Data Assimilation System (NLDAS) project. Journal of Geophysical Research 108: 8842.

4. Arulampalam MS, Maskell S, Gordon N, Clapp T (2002) A tutorial on particle filters for online nonlinear/non-Gaussian Bayesian tracking. Signal Processing, IEEE Transactions on 50: 174-188.

5. Shaman J, Karspeck A (2012) Forecasting seasonal outbreaks of influenza. Proc Natl Acad Sci USA 109: 20425-20430.

6. van Leeuwen PJ (2009) Particle Filtering in Geophysical Systems. Monthly Weather Review 137: 4089-4114.

7. Ionides EL, Breto C, King AA (2006) Inference for nonlinear dynamical systems. Proc Natl Acad Sci U S A 103: 18438-18443.

8. Ionides EL, Bhadra A, Atchade Y, King A (2011) Iterated filtering. Ann Stat 39: 1776-1802.

9. Andrieu C, Doucet A, Holenstein R (2010) Particle Markov chain Monte Carlo methods. Journal of the Royal Statistical Society: Series B (Statistical Methodology) 72: 269-342.

10. Evensen G (2008) Sequential data assimilation. Data Assimilation: The ensemble Kalman filter. 2nd ed. New York: Springer. pp. 27-46.

11. Anderson JL (2001) An Ensemble Adjustment Kalman Filter for Data Assimilation. Monthly Weather Review 129: 2884-2903.

12. Karspeck AR, Anderson JL (2007) Experimental Implementation of an Ensemble Adjustment Filter for an Intermediate ENSO Model. Journal of Climate 20: 4638-4658.

13. Anderson JL (2010) A non-Gaussian ensemble filter update for data assimilation. Monthly Weather Review 138: 4186-4198.

14. Sacher W, Bartello P (2008) Sampling errors in ensemble Kalman filtering. Part I: Theory. Monthly Weather Review 136: 3035-3049.

15. Anderson JL, Anderson SL (1999) A Monte Carlo Implementation of the Nonlinear Filtering Problem to Produce Ensemble Assimilations and Forecasts. Monthly Weather Review 127: 2741-2758.

16. Anderson JL (2009) Spatially and temporally varying adaptive covariance inflation for ensemble filters. Tellus A 61: 72-83.
